# Advanced renal cell carcinoma management: the Latin American Cooperative Oncology Group (LACOG) and the Latin American Renal Cancer Group (LARCG) consensus update

**DOI:** 10.1007/s00432-024-05663-z

**Published:** 2024-04-09

**Authors:** Andrey Soares, Fernando Sabino Marques Monteiro, Karine Martins da Trindade, Adriano Gonçalves e Silva, Ana Paula Garcia Cardoso, André Deeke Sasse, André P. Fay, André Paternò Castello Dias Carneiro, Antonio Machado Alencar Junior, Augusto César de Andrade Mota, Bruno Santucci, Daniel da Motta Girardi, Daniel Herchenhorn, Daniel Vilarim Araújo, Denis Leonardo Jardim, Diogo Assed Bastos, Diogo Rodrigues Rosa, Fabio A. Schutz, Fábio Roberto Kater, Felipe da Silva Marinho, Fernando Cotait Maluf, Fernando Nunes Galvão de Oliveira, Fernando Vidigal, Igor Alexandre Protzner Morbeck, Jose Augusto Rinck Júnior, Leonardo Atem G. A. Costa, Manuel Caitano Dias Ferreira Maia, Manuela Zereu, Marcelo Roberto Pereira Freitas, Mariane Sousa Fontes Dias, Milena Shizue Tariki, Pamela Muniz, Patrícia Medeiros Milhomem Beato, Paulo Sérgio Moraes Lages, Pedro Isaacsson Velho, Ricardo Saraiva de Carvalho, Rodrigo Coutinho Mariano, Sandro Roberto de Araújo Cavallero, Thiago Martins Oliveira, Vinicius Carrera Souza, Oren Smaletz, Stênio de Cássio Zequi

**Affiliations:** 1 Latin American Cooperative Oncology Group, Genitourinary Group (LACOG-GU), Av. Brigadeiro Faria Lima, Vila Olímpia, São Paulo, SP 4300 Brazil; 2 Hospital Israelita Albert Einstein, São Paulo, SP Brazil; 3 Centro Paulista de Oncologia/Oncoclínicas, São Paulo, SP Brazil; 4 Hospital Sírio-Libanês, Brasília, DF Brazil; 5 Oncologia D’Or, Fortaleza, CE Brazil; 6 Instituto do Câncer e Transplante de Curitiba/PR (ICTr Curitiba), Curitiba, PR Brazil; 7 Grupo SONHE de Campinas, Campinas, SP Brazil; 8 Escola de Medicina da Pontifícia, Universidade Católica do Rio Grande do Sul (PUCRS), Porto Alegre, RS Brazil; 9 Hospital Municipal Vila Santa Catarina, São Paulo, SP Brazil; 10 Hospital São Domingos, São Luís, MA Brazil; 11 Dasa Oncologia, Brasília, DF Brazil; 12 Hospital Universitário da Universidade Federal do Maranhão (UFMA), São Luís, MA Brazil; 13 Clínica AMO-DASA, Feira de Santana, BA Brazil; 14 Instituto Paulista de Cancerologia, São Paulo, SP Brazil; 15 Oncologia D’Or, Rio de Janeiro, RJ Brazil; 16 Hospital de Base de São José do Rio Preto/SP, São José do Rio Preto, São Paulo, SP Brazil; 17 Grupo Oncoclínicas, São Paulo, São Paulo, SP Brazil; 18 Hospital Sirio-Libanês de São Paulo, São Paulo, SP Brazil; 19 Grupo Oncoclínicas, Rio de Janeiro, RJ Brazil; 20 Beneficência Portuguesa de São Paulo, São Paulo, SP Brazil; 21 Grupo Oncoclínicas, Recife, PE Brazil; 22 Grupo Oncoclínicas, Salvador, BA Brazil; 23 Grupo Oncoclínicas, Brasília, DF Brazil; 24 AC Camargo Cancer Center, São Paulo, SP Brazil; 25 Hospital do Câncer Porto Dias, Belém, PA Brazil; 26 Santa Casa de Misericórdia de Porto Alegre, Porto Alegre, RS Brazil; 27 Centro Especializado de Oncologia de Florianópolis, Florianópolis, SC Brazil; 28 Universidade Federal de São Paulo (UNIFESP), São Paulo, SP Brazil; 29 Hospital Amaral Carvalho, Jaú, SP Brazil; 30 Hospital Moinhos de Vento, Porto Alegre, RS Brazil; 31 Johns Hopkins University, Baltimore, MD USA; 32 Hospital Adventista de Belém, Belém, PA Brazil; 33 Hospital São Rafael, Salvador, BA Brazil; 34 Instituto D’Or de Ensino e Pesquisa, Salvador, BA Brazil; 35 National Institute for Science and Technology in Oncogenomics and Therapeutic Innovation, AC Camargo Cancer Center, São Paulo, SP Brazil

**Keywords:** Renal cell carcinoma, Consensus, Tyrosine kinase inhibitor, Vascular growth factor receptor, Immunotherapy, Metastatic disease

## Abstract

**Purpose:**

Renal cell carcinoma is an aggressive disease with a high mortality rate. Management has drastically changed with the new era of immunotherapy, and novel strategies are being developed; however, identifying systemic treatments is still challenging. This paper presents an update of the expert panel consensus from the Latin American Cooperative Oncology Group and the Latin American Renal Cancer Group on advanced renal cell carcinoma management in Brazil.

**Methods:**

A panel of 34 oncologists and experts in renal cell carcinoma discussed and voted on the best options for managing advanced disease in Brazil, including systemic treatment of early and metastatic renal cell carcinoma as well as nonclear cell tumours. The results were compared with the literature and graded according to the level of evidence.

**Results:**

Adjuvant treatments benefit patients with a high risk of recurrence after surgery, and the agents used are pembrolizumab and sunitinib, with a preference for pembrolizumab. Neoadjuvant treatment is exceptional, even in initially unresectable cases. First-line treatment is mainly based on tyrosine kinase inhibitors (TKIs) and immune checkpoint inhibitors (ICIs); the choice of treatment is based on the International Metastatic Database Consortium (IMCD) risk score. Patients at favourable risk receive ICIs in combination with TKIs. Patients classified as intermediate or poor risk receive ICIs, without preference for ICI + ICIs or ICI + TKIs. Data on nonclear cell renal cancer treatment are limited. Active surveillance has a place in treating favourable-risk patients. Either denosumab or zoledronic acid can be used for treating metastatic bone disease.

**Conclusion:**

Immunotherapy and targeted therapy are the standards of care for advanced disease. The utilization and sequencing of these therapeutic agents hinge upon individual risk scores and responses to previous treatments. This consensus reflects a commitment to informed decision-making, drawn from professional expertise and evidence in the medical literature.

**Supplementary Information:**

The online version contains supplementary material available at 10.1007/s00432-024-05663-z.

## Introduction

Renal cell carcinoma (RCC) is the most common form of kidney cancer and represents 2% of all malignancies globally as well as in Brazil (Kidney Cancer Statistics 2023; WHO 2023; Padala et al. 2020). Although RCC mortality has decreased in developed countries over the years (Siegel et al. 2023), it is still a relevant cause of death in Brazil; from 1996 to 2019, 54,013 individuals died of RCC, resulting in 1.13 deaths per 100,000 persons/year in this country (Mourão et al. 2022).

RCC is an aggressive condition for half of patients with local or locally advanced disease relapse (Sameh et al. 2012; Correa et al. 2021). Fortunately, there are currently adjuvant treatment options with tyrosine kinase inhibitors (TKIs) and immune checkpoint inhibitors (ICIs) for patients at high risk of recurrence (Sharma et al. 2018).

Survival rates of RCC patients depend on stage, and 30% of patients in Brazil already have metastatic disease at diagnosis (Padala et al. 2020; Abreu et al. 2021; Bahadoram et al. 2022; Tran and Ornstein 2022). Treatment for metastatic disease has evolved in recent years due to the use of new immune combinations, such as ICIs or ICIs plus TKIs, which improve the response rate and overall survival (OS) (Rassy et al. 2020). However, identifying the best option in the first-line treatment setting as well as sequencing of systemic treatment in the current scenario are challenging.

This consensus provides an update on advanced RCC management from the previous expert panel recommendation of the Latin American Cooperative Oncology Group (LACOG) and the Latin American Renal Cancer Group (LARCG) published in 2020 (Soares et al. 2020).

## Methods

The LACOG–Genitourinary Group (LACOG-GU) and the LARCG gathered 72 specialists in medical oncology and urology, chosen according to their expertise in RCC, to discuss and vote on the best management of RCC in Brazil. The results are divided into two papers; this paper includes systemic therapy and was voted on and discussed by 34 oncologists.

One author (A.S.) prepared the questions based on a literature review considering the most recent advances in the last three years, as the last consensus was from 2020 (Soares et al. 2020). This questionnaire included data on adjuvant and neoadjuvant therapies, metastatic disease, brain metastasis, and osteoclast inhibitors; three other authors reviewed the questionnaire (F.S.M.M., K.T., and O.S.).

The oncologists received a link by email for individual access and response to a questionnaire comprising 112 multiple-choice questions. Each question had an “abstain” option for those who felt unable to choose an answer; this vote was not counted.

An independent facilitator assembled the results, and based on the Delphi consensus methods, the answers chosen by at least 75% of the participants were considered to constitute a consensus. A virtual meeting was held to discuss the questions that did not reach a consensus, and a second round of voting was carried out. Once again, the answers chosen by at least 75% of the participants were considered to constitute a consensus; otherwise, the most voted was considered to indicate a recommendation. The complete questionnaire and the results are available in the Supplementary Material.

The chosen answers were confronted with evidence from medical literature and graded according to an adapted classification from the Oxford Levels of Evidence classification (CEBM 2009) (Tables 1 and 2).

**Table 1:** Classification of the level of evidence, adapted from the Oxford classification 2009 (CEBM 2009)

Level of evidence	Characteristics
1a	Systematic review of randomized clinical studies
1b	Randomized clinical studies, nonrandomized clinical studies
2a	Systematic review of cohort studies
2b	Cohort studies or low-quality randomized clinical studies
3a	Systematic review of case‒control studies
3b	Case‒control studies
4	Case-series
5	Expert opinion

**Table 2:** Grade of recommendation, adapted from the Oxford Classification 2009 (CEBM 2009)

Grade	Characteristics
A	Consistent level 1 studies
B	Consistent level 2 or 3 studies or extrapolation from level 1 studies
C	Level 4 studies or extrapolation from level 2 or 3 studies
D	Level 5 evidence or troublingly inconsistent or inconclusive studies of any level

## Results

### Adjuvant treatment

The utilization of adjuvant therapy in RCC is limited due to the absence of a globally accepted standard. Recent discoveries indicate a potential breakthrough, as pembrolizumab has improved OS in patients with a high risk of recurrence (Broderick 2023). It is important to note that this information is derived from a press release, and the detailed data are not yet accessible. Notably, the positive outcome was not disclosed to the panel members before they voted.

The panel recommends adjuvant treatment for the majority of patients with node-positive clear cell renal cancer (ccRCC) (pTanyN + any grade) (recommendation; LE: 1b; GR: A) (Choueiri et al. 2021a) and only for a minority of patients with node-negative staging (≥ pT3N0 any grade) (recommendation; LE: 1b; GR: A) (Choueiri et al. 2021a; Powles et al. 2022). Patients with advanced ccRCC after nephrectomy and complete resection of all metastases (M1 NED—no evidence of disease), patients with locally advanced disease (pT3 or pT4), node-positive disease, high Fuhrman grade, or presenting sarcomatoid features have an increased risk of recurrence and seem to benefit the most from an adjuvant approach (Choueiri et al. 2021a; Leow et al. 2021). There are several nomograms for evaluating the risk of recurrence of RCC, but they showed low prediction according to the results of prospective evaluation in the ASSURE trial (Haas et al. 2016).

The agents that showed positive results for adjuvant treatment in selected patients with ccRCC ≥ pT3N0 of any grade included the ICI pembrolizumab and the vascular endothelial growth factor (VEGFR) inhibitor sunitinib (recommendation; LE: 1b; GR: A) (Choueiri et al. 2021a; Ravaud et al. 2016). However, in most cases, pembrolizumab should be used instead of sunitinib, considering the toxicity associated with sunitinib (Haas et al. 2017; Ravaud et al. 2016).

The panel recommends pembrolizumab as adjuvant therapy as follows:

- In node-positive ccRCC (pTanyN + any grade) (recommendation; LE: 1b; GR: A) (Choueiri et al. 2021a)
- In most ccRCC pTanyNanyM1 NEDs of any grade, there is also a high risk for disease recurrence (recommendation; LE: 1b; GR: A) (Choueiri et al. 2021a)
- In a minority of node-negative ccRCC patients (pT2N0 + grade 4 Fuhrman and/or sarcomatoid features and > pT3N0 any grade) (recommendation; LE: 1b; GR: A) (Choueiri et al. 2021a; Powles et al. 2022)

The KEYNOTE-564 trial was the first phase III trial to demonstrate the benefits of adjuvant treatment with pembrolizumab in patients with metastatic RCC and an intermediate to high risk of recurrence (Choueiri et al. 2021a; Powles et al. 2022). Pembrolizumab was associated with longer disease-free survival (DFS) (77.3% in the pembrolizumab group versus 68.1% in the placebo group, p = 0.002) (Choueiri et al. 2021a; Powles et al. 2022). Pembrolizumab has a manageable safety profile; however, it is important to follow patients because even though serious adverse events (AEs) are rare, they can be substantial and permanent. In the KEYNOTE-564 30-months follow-up analysis, 32% of patients in the pembrolizumab group versus 18% of patients in the placebo group presented grade 3 or worse AEs, with hypertension and increased alanine aminotransferase being the most frequent in the treated group; 21% of patients in the pembrolizumab group discontinued treatment due to AEs (Powles et al. 2022). A recent pooled analysis of phase III trials (n = 4125) evaluated the safety profile of pembrolizumab as an adjuvant treatment for different solid tumours, including RCC (Luke et al. 2022). The authors found grade 3–5 treatment-related AEs at 16.3% (the most common were diarrhoea, increased alanine aminotransferase, and colitis), discontinued treatment at 15.8%, immune-mediated AEs and infusion reactions at 36.9%, and 4 deaths (Luke et al. 2022).

The IMmotion010 trial evaluated the effectiveness and safety of atezolizumab compared to a placebo when used as adjuvant therapy in patients with RCC at high risk of recurrence following surgical resection (Bex et al. 2022). In this study, the DFS was 57.2 months (Hazard ratio (HR) 44.6, not evaluable (NE) ) for atezolizumab and 49.5 months (HR: 47.4, NE) for the placebo; however, these they were not significantly different between the groups (p = 0.495).

The results from the Checkmate 914 trial showed no benefit of nivolumab plus ipilimumab compared to placebo in the adjuvant treatment of localized RCC after nephrectomy that is associated with a high risk of recurrence (Motzer et al. 2023a). The median DFS for the combination therapy was not reached in the experimental group versus 50.7 months in the group treated with placebo (HR: 0.92; p = 0.53).

In the Prosper trial, the perioperative use of nivolumab versus observation in patients with ≥ T2 or TanyN + RCC resulted in similar recurrence-free survival (RFS) rates (HR: 0.97; 95% confidence interval (CI): 0.74, 1.28; p = 0.43) but no significant difference in OS (HR: 1.48; 95% CI: 0.89, 2.48; p = 0.93) (Allaf et al. 2022).

A recent meta-analysis evaluated different ICIs for adjuvant treatment of RCC (Monteiro et al. 2023). The authors identified four clinical trials (n = 3407) assessing pembrolizumab, atezolizumab, nivolumab, and ipilimumab plus nivolumab; however, the improvement in DFS was only statistically significant in subgroup analysis for PD-L1-positive patients (HR:0.72; 95% CI:0.55, 0.94), intermediate-high risk patients (HR:0.77; 95% CI:0.63, 0.94), and patients with a sarcomatoid component (HR:0.66; 95% CI:0.43, 0.99), highlighting the importance of individual evaluation when deciding about adjuvant therapy.

Surveillance emerges as a viable recourse in scenarios where the criteria for administering pembrolizumab are absent.

The use of sunitinib is limited to stage III onwards but supported by a lower level of evidence compared to pembrolizumab, with lack of benefits in OS and the presence of discordant outcomes between the S-TRAC trial and the ASSURE trial, associated with concerns regarding its toxicity profile. In the S-TRAC trial; 306 patients with T ≥ 3 and/or N + received the drug versus 304 treated with placebo, with a positive DFS result (its primary endpoint), p = 0.03, but no improvement in OS (Ravaud et al. 2016). In this trial, patients presented high toxicity and worsening of quality of life (Ravaud et al. 2016). In the ASSURE trial, conflicting DFS results were reported, and no improvement in OS (Haas et al. 2016).

Sunitinib should not be used as an adjuvant treatment in patients with non-ccRCC (consensus; LE: 1b; GR: A), as no benefit has been observed for disease with papillary, chromophobe, mixed, unclassified, or sarcomatoid features (Haas et al. 2016). In fact, no adjuvant treatment, including sunitinib or pembrolizumab, should be prescribed for patients with non-ccRCC.

When using sunitinib as adjuvant therapy, the panel recommends starting with a full dose (consensus; LE: 1b; GR: A) (Ravaud et al. 2016; Haas et al. 2016). The panel only recommends adjusting the dose of sunitinib in patients with toxicity grade 2 (recommendation; LE: 5; GR: D), whereas in patients with toxicity grade 3, the treatment should be interrupted (consensus; LE: 5; GR: D). Concerns have been raised about the toxicity of sunitinib and the frequency of related AEs in both clinical trials, independent of efficacy (Ravaud et al. 2016; Haas et al. 2016). Sixty-six percent of patients from the ASSURE trial and 63.4% of patients from the S-TRAC trial experienced grade 3 or higher AEs; 27.5% of patients with S-TRAC discontinued treatment due to AEs (Ravaud et al. 2016). A dose reduction partly alleviated the adverse effects in the ASSURE clinical trial (Haas et al. 2016). Nevertheless, 55% of patients who started treatment with lower doses experienced grade 3 AEs (Haas et al. 2016).

Everolimus in the EVEREST trial (Ryan et al. 2022), sorafenib in the SORCE trial (Eisen et al. 2020), pazopanib, in the PROTECT trial (Motzer et al. 2021a), and axitinib, in the ATLAS trial (Gross-Goupil et al. 2018) were also evaluated as adjuvant therapies in ccRCC for at least one year; however, they all failed to significantly improve DFS or OS.

**Table:** 

Adjuvant treatment
The agents for adjuvant treatment are pembrolizumab and sunitinib, with a preference for pembrolizumab
Pembrolizumab should be used as adjuvant treatment in node-positive ccRCC (pTanyN + any grade), in most ccRCC pTanyNanyM1 NED any grade, but in a minority of node-negative ccRCC pT2N0 + grade 4 Fuhrman and/or sarcomatoid features, and > pT3N0 any grade
No adjuvant treatment should be indicated in non-ccRCC, including sunitinib or pembrolizumab
Sunitinib should be started with a full dose; doses should be adjusted only if there is grade 2 toxicity; treatment should be discontinued in case of toxicity grade 3

### Neoadjuvant treatment

Neoadjuvant treatment for RCC in general is an exceptional approach (consensus; GR: 4; GR: C) (Thomas et al. 2009), and a multidisciplinary team decision should be made (consensus; LE: 5; GR: D) due to the lack of studies with adequate levels of evidence to support this indication routinely and the risk of toxicity.

The panel recommends neoadjuvant treatment in patients with both kidneys in place and unresectable locally advanced disease (consensus; LE: 4; GR: C) (Thomas et al. 2009) and for those with a solitary kidney who are not candidates for partial nephrectomy (consensus; LE: 5; GR: D).

The panel does not recommend neoadjuvant treatment in patients with both kidneys in place and resectable locally advanced disease (consensus LE: 5; GR: D) or for those with a solitary kidney and eligible for partial nephrectomy (consensus; LE: 5; GR: D), as surgery remains the best curative option for local disease, and no evidence of benefit of neoadjuvant treatment is currently available for these patients.

The panel recommends neoadjuvant treatment with ICIs + TKIs (consensus; LE: 1b; GR: B). According to the Checkmate 214, Checkmate 9ER and CLEAR studies evaluating the Response Evaluation Criteria in Solid Tumours (RECIST) in primary lesions in patients who did not undergo nephrectomy, those who received monotherapy (sunitinib) had a lower response in the primary tumour (Motzer et al. 2021b, 2023b; Burotto et al. 2023). These studies did not evaluate neoadjuvant treatment, but they indirectly evaluated the response of the primary tumour. TKI alone is the choice for those not able to receive ICI + TKI; in this case, the panel recommends cabozantinib (recommendation; LE: 1b; GR: B). Cabozantinib is the TKI most commonly used as a monodrug for intermediate/poor risk advanced disease. The recommendation for its use in neoadjuvant treatment is based on the extrapolation of results in advanced disease (Choueiri et al. 2017).

There was no preference among the drugs chosen for combination ICI + TKI therapy with an OS objective (pembrolizumab + axitinib, pembrolizumab + levantinib, avelumab + axitinib, or nivolumab + cabozantinib) (recommendation; LE: 5; GR: D).

Specifically, for unresectable cases, the panel recommends TKI + ICI (recommendation; LE: 5; GR: D).

**Table:** 

Neoadjuvant treatment
Neoadjuvant treatment is an exceptional approach, even in initially unresectable cases, after multidisciplinary team discussion
Neoadjuvant treatment can be indicated for patients with both kidneys and irresectable locally advanced disease or for patients with a solitary kidney who are not candidates for partial nephrectomy
Neither patients with both kidneys and resectable locally advanced disease nor patients with solitary kidney and eligible for partial nephrectomy should receive neoadjuvant therapy
The combination of ICI + TKI is the choice for neoadjuvant treatment, without any preference among the combos with OS advantage
TKI alone (cabozantinib) is the choice for those not able to receive ICI

### Advanced renal cell carcinoma management

#### First-line therapy for ccRCC

The International Metastatic Database Consortium (IMDC) risk score is important for defining the first-line systemic treatment of metastatic ccRCC (consensus; LE: 5; GR: D), as it can correlates survival to treatment choice (Heng et al. 2013, 2009). The IMDC risk score is a relevant prognostic tool for patients with metastatic RCC that divides patients into favourable, intermediate, or poor-risk groups according to the time from diagnosis to treatment > or < 1 year, Karnofsky performance score > or < 80%, and the presence or absence of anaemia, hypercalcaemia, thrombophilia, or neutrophilia (Heng et al. 2013).

PD-L1 expression is not essential for defining first-line systemic treatment for metastatic ccRCC (consensus; LE: 1b; GR: A). It seems to be associated with worse prognosis in RCC (Lu et al. 2020); however, studies have not clearly identified a correlation between treatment efficacy and PD-L1 status (Motzer et al. 2022a, 2021c; Rini et al. 2019a).

The first-line treatment for patients with metastatic ccRCC and favourable IMDC risk should be based on ICIs (consensus; LE: 1b; GR: A) (Rini et al. 2023; Motzer et al. 2019; Choueiri et al. 2021b) but not monotherapy; rather, it should be combined with TKIs (consensus; LE: 1b; GR: A) (Rini et al. 2023; Motzer et al. 2019; Choueiri et al. 2021b). When using TKI monotherapy as the first-line treatment in patients with IMDC favourable risk for metastatic ccRCC who cannot receive or tolerate ICI therapy, there is no preference for pazopanib or sunitinib, but most experts prefer pazopanib (recommendation; LE:1b; GR: A) (Motzer et al. 2013). Pazopanib provides progression-free survival (PFS) and OS similar to those of sunitinib but has a better safety profile and higher quality-of-life scores (Motzer et al. 2013).

For intermediate risk patients, the panel recommends ICI-based therapy (consensus; LE: 1b; GR: A) (Rini et al. 2023; Motzer et al. 2019, 2018, 2021c; Choueiri et al. 2021b), without preference for ICI + ICI or ICI + TKI (recommendation; LE: 5; GR: D). When using TKIs as monotherapy and a first-line treatment in patients at intermediate risk who cannot receive or tolerate ICIs, the panel recommends cabozantinib (consensus; LE: 1b; GR: B) (Choueiri et al. 2017). In a phase II trial, cabozantinib was shown to have superior PFS and a higher objective response rate (ORR) than sunitinib as an initial targeted therapy for patients with metastatic RCC and poor or intermediate risk (8.2 versus 5.6 months, HR:0.66, 95% CI:0.46, 0.95; p = 0.012), but there was no statistically significant difference in OS (30.3 months with cabozantinib versus 21.8 months with sunitinib; HR:0.80; 95% CI:0.50, 1.26) (Choueiri et al. 2017).

Patients at poor risk should be treated with ICI-based therapy (consensus; LE: 1b; GR: A) (Rini et al. 2023; Motzer et al. 2019, 2018, 2021c; Choueiri et al. 2021b) without any preference for ICI + ICI or ICI + TKI (recommendation; LE: 5; GR: D).

When using an ICI + TKI combination as a first-line treatment in patients with metastatic ccRCC, there is no preference for pembrolizumab + axitinib, pembrolizumab + levantinib, or nivolumab + cabozantinib (consensus; LE: 5; GR: D).

In the KEYNOTE-426 trial, which included a median follow-up of 67.2 months, pembrolizumab plus axitinib was superior to sunitinib in terms of OS (47.2 versus 40.8 months; HR:0.84; 95% CI:0.71, 0.99) and PFS (15.7 versus 11.1 months; HR:0.69; 95% CI:0.59, 0.81) (Rini et al. 2023). The superiority of the combination was observed, especially in the intermediate- and poor-risk groups (Rini et al. 2023).

In a phase III randomized clinical trial evaluating avelumab plus axitinib versus sunitinib, the combination of avelumab plus axitinib provided longer PFS (13.9 versus 8.5 months with sunitinib, p < 0.0001) (Haanen et al. 2023). For each subgroup of IMDC risk, the HR for disease progression or death and the ORR also supported this association (Haanen et al. 2023). However, the OS data are still immature, the median OS for the combination therapy was not reached (95% CI: 42.2 months-NE versus 37.8 months 95% CI: 31.7%-NE; p = 0.0116), and the study is still ongoing.

The Checkmate 214 study comparing nivolumab plus ipilimumab versus sunitinib showed that the combination is beneficial for intermediate- and poor-risk ccRCC patients (Motzer et al. 2022b). The combination was associated with improved median OS (55.7 vs. 38.4 months, p < 0.0001) and 5-year PFS (31% vs. 11%, p = 0.0628) as well as higher ORR (42% vs. 27%, p < 0.0001). The study was not designed to evaluate efficacy in favourable-risk patients.

In the Checkmate 9ER study with 3-year follow-up, nivolumab plus cabozantinib presented higher median 12-month OS than sunitinib did (49.5 versus 35.5 months, p = 0.0014), and the combination was associated with better PFS (16.6 versus 8.4 months, p < 0.001) and higher ORR (55.7% (95% CI: 50, 61) vs. 28.4% (95% CI: 24, 34)) (Burotto et al. 2023).

According to the CLEAR study, after 4 years of follow-up, patients treated with levantinib and pembrolizumab had longer PFS (HR:0.47; 95% CI:0.38, 0.57; p < 0.0001), higher OS (HR:0.79; 95% CI:0.63, 0.99; p = 0.0424), and higher ORR (71.3% versus 36.7%, relative risk = 1.94; 95% CI:1.67, 2.26) than did patients treated with sunitinib, especially in intermediate- and poor-risk groups of patients (Motzer et al. 2023b).

The COSMIC-313 study evaluated the combination of cabozantinib + nivolumab + ipilimumab versus placebo + nivolumab + ipilimumab in previously untreated patients with ccRCC and intermediate (75%) or poor (25%) risk (Choueiri et al. 2023). The triple combination was associated with longer 12-month PFS (57% versus 49%; HR:0.73; 95% CI:0.57, 0.94; p = 0.01). However, OS evaluation is still ongoing.

The combinations of ICI and TKI have similar toxicity profiles that persist for longer due to the use of TKI and the association of ICI and ICI shows greater toxicity at the beginning of treatment. ICI and TKI have more discontinuation, but less use of high-dose corticosteroids when compared to nivolumab with ipilimumab (Rini et al. 2023; Haanen et al. 2023; Motzer et al. 2022b, 2023b; Burotto et al. 2023; Choueiri et al. 2023). ICI and TKI do not improve quality of life (QoL); only in the nivolumab and cabozantinib study QoL was it better than sunitinib but did not achieve a benefit in predetermined QoL in the tresholds, as well as the nivolumab /ipilimumab study (Rini et al. 2023; Haanen et al. 2023; Motzer et al. 2022b, 2023b; Burotto et al. 2023; Choueiri et al. 2023).

The panel recommends ICI + TKIs for patients with metastatic ccRCC and high burden/symptomatic disease (consensus; LE: 1a; GR: B) based on a high response rate and low progressive disease rate as the best response associated with this combination (Rini et al. 2023; Motzer et al. 2019; Choueiri et al. 2021b).

Patients with metastatic ccRCC and sarcomatoid/rhabdoid features should be treated with ICI + ICI (consensus; LE: 2b; GR: B) (Tannir et al. 2021). According to our evaluation of this subgroup of Checkmate 214 patients, which included 139 patients with sarcomatoid features, treatment with nivolumab combined with ipilimumab was superior to treatment with sunitinib, with improved OS (HR:0.45; 95% CI:0.3, 0.7; p = 0.0004) and longer PFS (26.5 versus 5.1 months; HR:0.54; 95% CI:0.33, 0.86; p = 0.0093) (Tannir et al. 2021). The combination of ICI + TKI also shows benefit in patients with sarcomatoid features (Choueiri et al. 2021c; Rini et al. 2019b; Motzer et al. 2021b). Avelumab plus axitinib was associated with longer PFS (7 months (95% CI: 5.3, 13.8 months) than sunitinib (4 months (95% CI:2.7, 5.7 months) ) and better ORR (46.8% (95% CI:32.1%, 61.9%) versus 21.3%, (95% CI:11.9%, 33.7%), respectively) (Choueiri et al. 2021c). Treatment with pembrolizumab plus axitinib improved the OS of patients with sarcomatoid features compared with sunitinib alone (HR:0.58; 95% CI:0.21, 1.59; 12-month rate: 83.4% vs. 79.5%) and provided longer PFS (HR:0.54; 95% CI:0.29, 1.00; median not reached vs. 8.4 months) (Rini et al. 2019b). The ORR was also superior with the combination: 58.8% (95% CI:44.2, 72.4) versus 31.5% (95% CI:19.5, 45.6) with sunitinib (Rini et al. 2019b). Treatment with nivolumab plus cabozantinib also improved PFS, OS, and ORR in patients with sarcomatoid features, as it is superior to treatment with sunitinib alone (Motzer et al. 2021b). The PFS for patients treated with the combination was 10.9 months versus 4.2 months with sunitinib (HR:0.39; 95% CI:0.22, 0.70). OS was not reached with the combination therapy and was 19.7 months with sunitinib (HR:0.36; 95% CI:0.16, 0.82). The ORR was 55.9% for nivolumab plus cabozantinib and 22.0% for sunitinib.

Patients with autoimmune disease should be evaluated before using TKIs; all treatment should be ICI based in individuals with formal contraindication to TKI.

With the current data on combinations of antiangiogenic agents and immunotherapy or ICI-ICI therapy, the panel members believe that high-dose IL2 no longer plays a role in the management of advanced RCC (consensus; LE: 1b; GR: B) due to the low rate of complete response and high risk of severe toxicity (Fyfe et al. 1995).

**Table:** 

First-line therapy
The IMDC is important to define the first-line systemic treatment of metastatic ccRCC
The PD-L1 expression is not essential to define the first-line systemic treatment of metastatic ccRCC
The first-line treatment for patients with metastatic ccRCC and IMDC favourable risk should be based on ICI combined with TKI; for patients who cannot receive ICI, sunitinib or pazopanib are indicated, but preference is given to pazopanib due to its safety profile and higher quality-of-life scores
The first-line treatment for patients with metastatic ccRCC and IMDC intermediate risk, the panel recommends ICI-based treatment, without preference between ICI + ICI or ICI + TKI; for patients who cannot receive ICI, cabozantinib is indicated
The first-line treatment for patients with metastatic ccRCC and IMDC poor risk, the panel recommends ICI-based without preference between ICI + ICI or ICI + TKI
When using the combination of ICI + TKI, there is no preference among pembrolizumab + axitinib, pembrolizumab + levantinib, or nivolumab + cabozantinib
Patients with metastatic ccRCC and high burden/symptomatic disease should be treated with ICI + TKI
Patients with metastatic ccRCC and sarcomatoid/rhabdoid features should be treated with ICI + ICI
High-dose IL2 no longer has a role in the treatment of advanced RCC

#### First-line therapy: progression after adjuvant therapy

The treatment choice for patients who progress after adjuvant treatment will depend on the timing of their progression and their IMDC risk. Disease progression within six months may indicate drug resistance (Yang et al. 2023). When patients with metastatic ccRCC progress within six months after adjuvant treatment with pembrolizumab, the panel recommends the TKI cabozantinib as the first-line treatment for intermediate-risk (consensus; LE: 5; GR: D) and poor-risk (recommendation; LE: 5; GR: D) patients. According to evidence from the CONTACT 03 trial, the sequential use of ICI during or after progression within 6 months is not recommended (Pal et al. 2023). For patients who receive sunitinib as adjuvant treatment and progress within six months, the panel recommends ICI-based therapy (consensus; LE: 5; GR: D) without preference for ICI + ICI or ICI + TKI (recommendation; LE: 5; GR: D). When a TKI is used as a first-line treatment, cabozantinib is indicated (consensus; LE: 1b; GR: B) (Choueiri et al. 2017).

For patients with intermediate or poor risk of progression > 6 months and < 12 months after adjuvant pembrolizumab, the panel recommends ICIs in combination with TKIs (consensus; LE: 5; GR: D). For patients who received adjuvant sunitinib, there is no preference for ICI + ICI or ICI + TKI (consensus; LE: 5; GR: D). When using TKIs, the panel recommends cabozantinib (consensus; LE: 1b; GR: B) (Choueiri et al. 2017).

Patients who progress after 12 months of adjuvant pembrolizumab or sunitinib should be treated with ICIs with no preference for ICI + ICI or ICI + TKI (consensus; LE: 5; GR: D).

**Table:** 

First-line therapy: progression after adjuvant therapy
Progression within 6 months
Patients with metastatic ccRCC and intermediate or poor risk progressing within six months after adjuvant treatment with pembrolizumab should be treated with TKI (cabozantinib); if using ICI-based, it should be combined with TKI
Patients with metastatic ccRCC and intermediate or poor risk progressing within six months after adjuvant treatment with sunitinib should be treated with ICI-based therapy without preference between ICI + ICI or ICI + TKI. When ICI is not available or contraindicated, the choice for TKI is cabozantinib
Progression > 6 months and < 12 months
Patients with metastatic ccRCC and intermediate or poor risk progressing > 6 months and < 12 months after adjuvant pembrolizumab should be treated with the combination ICI + TKI
Patients with metastatic ccRCC and intermediate or poor risk progressing > 6 months and < 12 months after adjuvant sunitinib should be treated with ICI + ICI or ICI + TKI; when ICI is not available or contra-indicated, the choice of TKI is cabozantinib
Progression after 12 months
Patients progressing after 12 months of adjuvant pembrolizumab or sunitinib should be given ICI-based treatment with ICI + ICI or ICI + TKI

#### Second-line therapy for ccRCC

Even though standard care involving ICIs as first-line therapy has been established for RCC, progression after initial therapy can still be observed in the majority of patients that will eventually require subsequent therapy (Barata et al. 2018; Lee et al. 2021a). Optimal sequencing is necessary to improve outcomes (Barata et al. 2018). Commonly used agents for second- and later-line therapies include VEGFR-TKIs, mammalian target of rapamycin (mTOR) inhibitors, and ICIs (Shah et al. 2019; Motzer et al. 2015a; Choueiri et al. 2016). The most efficient way to sequence these agents is still an object of clinical trials.

For patients whose disease progresses after the combination of nivolumab and ipilimumab, the panel recommends TKI as a second-line treatment (consensus; LE: 2b; GR: B) (Auvray et al. 2019; Shah et al. 2019) with cabozantinib (consensus; LE: 1a; GR: B) (Heo et al. 2021). For patients who progress after combining an ICI and TKI in the first line, the panel recommends therapy with a previously unused TKI agent (consensus; LE: 5; GR: D).

There is evidence showing the clinical benefit of using TKIs as a second-line treatment after first-line treatment with the ICI combination of nivolumab plus ipilimumab (Auvray et al. 2019; Shah et al. 2019). A meta-analysis classified cabozantinib as the most efficient agent in this context (Heo et al. 2021). The ongoing phase II CaboPoint trial was designed to evaluate cabozantinib as a second-line agent (Albiges et al. 2023a, b). The results from the interim analysis, with 88 patients and at least 3 months of follow-up, showed the preliminary efficacy of cabozantinib, with an ORR of 29.5% (Albiges et al. 2023a, b).

When there is progression after first-line TKI therapy, nivolumab should be used in most cases (recommendation; LE: 1b; GR: A) (Motzer et al. 2015a, 2020), while cabozantinib should be used in select patients (consensus, LE: 1a; GR: A) (Wiecek and Karcher 2016; Amzal et al. 2017) as a second-line agent, especially those with a worse prognosis (Wiecek and Karcher 2016). The CheckMate 025 clinical trial (Motzer et al. 2015a) evaluated patients with advanced ccRCC receiving nivolumab after failure of first-line treatment with TKIs. The trial and follow-up (Motzer et al. 2020) showed nivolumab to be an effective and adequate drug for improving OS and reducing AEs compared to everolimus. According to its extended follow-up assessment, nivolumab was superior to everolimus, with a 7-year OS of 18% versus 11% (HR:0.74; 95% CI:0.63, 0.86) and a 7-year PFS of 4% versus 0% (HR:0.84; 95% CI:0.72, 0.99) (Escudier et al. 2022). Based on the findings from a meta-analysis, there seems to be no difference between nivolumab and cabozantinib (Wiecek and Karcher 2016); however, real-life data suggest that cabozantinib may be more effective in selected patients, such as those with bone metastasis and progression, during the use of sunitinib. (Santoni et al. 2022). Moreover, another meta-analysis showed the superiority of cabozantinib over other second-line options, such as everolimus, axitinib, sorafenib, and nivolumab (Amzal et al. 2017).

#### Third-line therapy for ccRCC

In the case of disease progression despite first-line therapy with TKIs and second-line systemic treatments, third-line targeted therapy might offer benefits; however, less than 10% of patients typically receive treatment in this scenario (Ishihara et al. 2018; Santoni et al. 2023). The limited utilization of third-line therapy can be attributed to the insufficient data available regarding its effectiveness and the absence of established guidelines to support its application. The panel recommends the combination of lenvatinib and everolimus as third-line treatment in advanced RCC patients based on clinical experience and the limited data available (Motzer et al. 2015b) in the following cases:

- Patients who progressed after combination immunotherapy treatment (ICI + ICI or ICI + TKI) and a second-line TKI treatment (consensus, LE: 5; GR: D)
- Progression of disease following treatment with cabozantinib as first-line therapy and immunotherapy with nivolumab as second-line therapy (consensus, LE: 5; GR: D)

On the other hand, for patients who progress after treatment with VEGFR inhibitors and everolimus, the panel recommends nivolumab (consensus; LE: 5; GR: D).

According to available clinical trials, everolimus extends PFS in RCC patients after progression following one or more systemic treatments (Motzer et al. 2008; Calvo et al. 2012), and it is an effective and well-tolerated agent (Amato and Stepankiw 2013). A possible alternative could be the combination of everolimus and lenvatinib (Motzer et al. 2015b). A recent systematic review evaluated lenvatinib with everolimus, cabozantinib, and nivolumab as second- or third-line therapies after progression following VEGFR-targeted treatment (Karner et al. 2019). It was concluded that each option improved PFS and OS, and the combination of lenvatinib and everolimus was the most favourable option. In contrast, nivolumab was less effective but safer than the other agents, with fewer grade 3 and 4 AEs (Karner et al. 2019).

**Table:** 

Second- and third-line therapies
Patients progressing after nivolumab + ipilimumab should receive cabozantinib
Patients progressing after ICI + TKI should receive previously unused TKI agent
The combination of lenvatinib and everolimus as third-line treatment is indicated for patients who progress after combo immunotherapy treatment (ICI + ICI or ICI + TKI) and a TKI in second-line treatment
The combination of lenvatinib and everolimus as third-line treatment is indicated for patients presenting progression after cabozantinib as first-line therapy and immunotherapy with nivolumab in the second line
Patients who progress after treatment with VEGF inhibitors and everolimus should receive nivolumab

#### First-line treatment for metastatic disease (non-cc histology)

Clear cell (cc) is the most common histological subtype of RCC, followed by papillary (approximately 10% of cases) and chromophobe (approximately 5%) RCC (Padala et al. 2020). Therapy sequencing for non-ccRCC has been scarcely studied. Phase III clinical trials are lacking, and non-ccRCC is usually evaluated as a subgroup next to ccRCC, leaving assessment of efficient agents and their use in sequence unsettled (Vera-Badillo et al. 2015; Osterman and Rose 2020).

In several studies, scholars encourage enrolling patients in clinical trials (Osterman and Rose 2020; Fernández-Pello et al. 2017; Tannir et al. 2012), and they promote the need for future research to develop new therapy concepts, such as ICIs following progression after first-line therapy in the case of non-cc histology (Osterman and Rose 2020; Fernández-Pello et al. 2017; Ciccarese et al. 2017; Vera-Badillo et al. 2015). Patients with non-ccRCC seem to benefit less from targeted systematic therapy than do those with ccRCC, as response rates are lower and PFS and OS are shorter (Vera-Badillo et al. 2015). Monotherapy with TKIs has been found to be effective for non-ccRCC patients, either as first- or second-line agents (Pal et al. 2021; Jung et al. 2018; Sneed et al. 2019).

The first-line treatment for most papillary tumours is cabozantinib (recommendation; LE: 2a; GR: B) (Pal et al. 2021). The SWOG PAPMET was a randomized phase II trial evaluating cabozantinib, crizotinib, and savolitinib versus sunitinib as first- or second-line treatments in patients with metastatic papillary RCC who had one line of preceding anticancer therapy (n = 152) (Pal et al. 2021). Patients treated with cabozantinib had longer PFS than those treated with sunitinib (9 months versus 5.6 months; HR for progression or death:0.60 (95% CI:0.37, 0.97; p = 0.019)), and no improvement in PFS was observed with crizotinib or savolitinib. Another phase II single-arm trial involving a smaller population without previous treatment showed an ORR of 52.9% with lenvatinib plus pembrolizumab (Albiges et al. 2023a, b). Cabozantinib and nivolumab in papillary RCC patients with no or maximally one line of treatment before the trial and a 34-month median follow-up showed a median PFS of 13.1 months, OS of 28 months, and 54%-ORR (Lee et al. 2023). Nivolumab with ipilimumab does not improve outcomes of patients with non-cc RCC (Tykodi et al. 2022; Kilari et al. 2021; Gupta et al. 2020).

Lenvatinib in combination with everolimus should be used for most chromophobe tumours (consensus; LE: 2b; GR: B) (Hutson et al. 2021). In a phase II study, 29% (n = 9) of patients with previously untreated chromophobe tumours had a 44% partial response, and 33% had stable disease with lenvatinib in combination with everolimus with a clinical benefit rate of 78% (Hutson et al. 2021).

Fumarate hydratase (FH) mutant tumours are a rare non-ccRC subtype with poor prognosis (Lindner et al. 2022). Bevacizumab + erlotinib should be used for most FH mutant tumours (consensus; LE: 2b; GR: B) (Srinivasan et al. 2020). The AVATAR trial, a phase II clinical trial, included patients with hereditary leiomyomatosis and papillary RCC, a familial cancer syndrome caused by an enzyme FH mutation, versus patients with sporadic papillary RCC and at most two previous treatments with VEGFR inhibitor agents (Srinivasan et al. 2020). Patients with FH-deficient advanced papillary RCC showed that treatment with bevacizumab + erlotinib was superior in this population, with longer PFS (21.1 months (95% CI: 15.6, 26.6) versus 8.7 months (95% CI: 6.4, 12.6) in the sporadic group) and higher ORR (64% (95% CI: 49, 77) versus 37% (95% CI: 24, 52)) (Srinivasan et al. 2020).

Microphthalmia transcription factor (MiT) family aberration-associated RCC is a rare condition affecting young patients, and these tumours are usually misdiagnosed as cc, papillary, or chromophobe RCC (Caliò et al. 2019). ICI + TKI should be used for most MiT family translocation tumours (consensus; LE: 5; GR: D).

Platinum-based chemotherapy should be used for the majority of collecting duct tumours (consensus; LE: 4; GR: C) (Dason et al. 2013) and for medullary tumours (consensus; LE: 2b; GR: B) (Iacovelli et al. 2015). Collecting duct and medullary tumours are rare, aggressive and generally present with metastatic disease (Dason et al. 2013; Blas et al. 2019). Data on platinum-based therapy for collecting duct tumours show an ORR of 26% versus no response to immunotherapy (Dason et al. 2013). Medullary tumours are refractory to targeted therapies (Beckermann et al. 2017). Platinum-based chemotherapy is associated with longer PFS (8.0 versus 1.0 months; p = 0.028) and OS (12.0 versus 7.0 months; p = 0.031) in patients with advanced disease than treatment with topoisomerase inhibitors, without significant differences between cisplatin, paclitaxel and gemcitabine regimens or methotrexate, vinblastine, doxorubicin, and cisplatin regimens (Iacovelli et al. 2015).

ICI + TKI should be used for most unclassified tumours associated with advanced disease (consensus; LE: 1b; GR: B) (Albiges et al. 2023a). In a phase II trial, pembrolizumab plus lenvatinib had an ORR of 52% for unclassified tumours and a 90% disease control rate, defined as partial response, complete response and stable disease (Albiges et al. 2023a). Nevertheless, the combination of nivolumab and ipilimumab has limited efficacy for unclassified tumours, as a retrospective study showed an ORR of 0% in this subgroup, a median OS of 7.4 months, and a PFS of 2.8 months (Alhalabi et al. 2022). Nivolumab alone provided an ORR of 44.4%, with a median PFS of 5.5 months (Chahoud et al. 2020), and pembrolizumab alone provided an ORR of 30.8% (Lee et al. 2021b).

**Table:** 

First-line treatment for metastatic non-ccRCC
First-line treatment for most papillary tumours is cabozantinib
Lenvatinib + everolimus should be used for most chromophobe tumours
Bevacizumab + erlotinib should be used for most FH mutant tumours
ICI + TKI should be used for most MiT family translocations
Platinum-based chemotherapy should be used for the majority of collecting ducts tumours and medullary tumours
ICI + TKI should be used for most unclassified tumours

#### Second-line treatment for metastatic disease (non-cc histology)

The panel recommends sunitinib, pazopanib, or cabozantinib (consensus; LE: 2b; GR: B) for patients with non-ccRCC after first-line treatment with mTOR inhibitors (Knox et al. 2017; Tannir et al. 2016).

The RECORD-3 phase II trial showed improvements in PFS and OS with sunitinib as a second-line treatment for patients previously treated with everolimus, even though longer OS and longer PFS were observed for the group treated with sunitinib followed by everolimus (OS: 29.5 months versus 22.4 months; PFS: 22.2 months versus 21.7 months) (Knox et al. 2017). Sunitinib, as a second-line treatment, was associated with PFS rates similar to those of everolimus (1.8 months versus 2.8 months, respectively; p = 0.6) in another phase II trial in which everolimus and sunitinib were evaluated for the treatment of metastatic non-ccRCC in a crossover design (Tannir et al. 2016).

The panel recommends the combination of bevacizumab and erlotinib following treatment with TKIs for the majority of papillary tumours (recommendation; LE: 2b; GR: B). Although clinical data on therapeutic regimens or second-line agents for non-ccRCC have yet to be obtained, monotherapies seem less effective for non-ccRCC than for ccRCC. In the AVATAR trial, bevacizumab and erlotinib provided an ORR of 51% and a median PFS of 14.2 months for patients with papillary RCC treated with at most two previous treatments comprising VEGFR inhibitors (Srinivasan et al. 2020).

The panel recommends combined therapy with ICIs in patients with sarcomatoid patterns (consensus; LE: 5; GR: D). In the case of tumours with a sarcomatoid pattern, the available evidence implies that ICIs can be a beneficial choice (Hanif et al. 2019; Raychaudhuri et al. 2017). A retrospective review of 30 patients (Hanif et al. 2019) and a case report (Raychaudhuri et al. 2017) showed positive responses to ICIs and improved outcomes.

**Table:** 

Second-line treatment for metastatic non-ccRCC
Sunitinib, pazopanib, or cabozantinib should be used for patients with non-ccRCC after first-line treatment with mTOR inhibitors
The combination of bevacizumab and erlotinib should be used following treatment with TKI for the majority of papillary tumours
The combined therapy of ICI should be used in patients with sarcomatoid patterns

### Active surveillance

Active surveillance (AS) refers to deferring active systemic treatment for selected patients to reduce toxicity without compromising efficacy (Harrison et al. 2021; Rini et al. 2016; Kushnir et al. 2021). This approach is suitable as an initial strategy for a carefully selected group of asymptomatic patients with a slow-growing, relatively limited disease volume (Harrison et al. 2021; Rini et al. 2016; Kushnir et al. 2021; Tenold et al. 2020).

Favourable-risk IMDC patients are possible candidates for AS (consensus; LE:2b; GR: A) (Rini et al. 2016), whereas intermediate- or poor-risk IMDC patients should not be selected for AS (consensus; LE: 4; GR: C) (Bimbatti et al. 2018).

The panel recommends AS as a strategy in a minority of patients with one metastatic site (recommendation; LE:1b; GR: B) (Harrison et al. 2021; Rini et al. 2016) but does not recommend it in patients with ≥ 2 metastatic sites (consensus; LE: 1b; GR: B) (Rini et al. 2016). The number of metastatic sites impacts the success of AS treatment (Harrison et al. 2021; Rini et al. 2016). Further factors can also influence the benefits of AS. A prospective phase II trial showed that a Karnofsky score of less than 100% or liver metastasis was associated with shorter surveillance periods and earlier initiation of systemic therapy (Rini et al. 2016).

Patients with lung metastasis or lymph node metastasis may be candidates for AS, whereas in the case of liver, brain, or bone metastasis, AS is not recommended (consensus; LE: 5; GR: D).

Several studies have concluded that patients with a low extrarenal disease burden and the absence of visceral crises could be ideal candidates for AS (Kushnir et al. 2021; Bimbatti et al. 2018; Park et al. 2014; Nizam et al. 2020). On the other hand, the panel does not recommend AS for patients with symptoms from metastatic RCC (consensus; LE: 4; GR: C) (Bimbatti et al. 2018).

**Table:** 

Active surveillance
IMDC favourable-risk patients are possible candidates for active surveillance
IMDC intermediate or poor-risk patients should not be selected for active surveillance
Active surveillance is suitable for a minority of patients with one metastatic site and not suitable for those ≥ 2 metastatic sites
Patients with lung metastasis or lymph-node metastasis may be candidates for active surveillance
Patients with liver, brain, or bone metastasis are not candidate for active surveillance

### Brain metastasis

Approximately 2–15% of patients with metastatic RCC have brain metastasis (Sun et al. 2019; Nieder et al. 2011). With the advance of new therapies and improved survival, the incidence of brain metastasis has increased (Matsui 2020), and the prognosis is generally poor (Levitin et al. 2020). A suitable therapeutic approach—local or systemic—is vital for longitudinal disease control and preservation of quality of life.

In the case of metastatic ccRCC with limited brain metastasis (1–3 lesions), surgery could be an option; however, the panel recommends stereotactic body radiation therapy (SBRT) for most patients due to minor morbidities (consensus; LE: 2b; GR: B) (Meyer et al. 2018). SBRT is effective and safe in patients with oligometastatic and oligoprogressive metastatic RCC. It can postpone the introduction of systematic therapy (Meyer et al. 2018) and be used as a therapeutic option instead of surgery (Wei et al. 2020).

In the case of several brain metastases, the panel recommends whole-brain radiotherapy (WBRT) as local therapy for most patients (consensus; LE: 2b; GR: B) (Hansen et al. 2019). WBRT is another therapeutic option that was, for a long time, the most common treatment for brain metastasis (Ippen et al. 2015). WBRT is an effective approach; however, SBRT is prioritized due to its rare effect on cognitive functions compared to WBRT, which is often associated with neurocognitive decline as an AE (Nabors et al. 2014). Consequently, practitioners currently prefer SBRT and reserve WBRT for patients with poorer prognoses, multiple brain metastases (> 10–15 lesions), or progressive, recurrent tumours (Hasanov et al. 2022). Individual decisions should consider factors such as the patient's expected lifespan (Hansen et al. 2019). Despite the poor prognosis of this disease, patients who receive WBRT experience a survival benefit from this treatment (Cannady et al. 2004).

In patients with metastatic ccRCC and limited brain metastasis (1–3 lesions) for whom no or low-dose steroids are needed, the panel recommends combination therapy comprising ICIs as systemic therapy (recommendation; LE: 2b; GR: B) (Emamekhoo et al. 2022). In systemic therapy for brain metastasis, corticosteroids and ICIs are essential components; however, their interaction seems paradoxical. Treatment with a combination of these two drugs was associated with worse OS (Jessurun et al. 2021). On the other hand, the CheckMate 920 phase III b/4 clinical trial suggested that ICI combinations may benefit patients with metastatic RCC involving brain metastasis (Emamekhoo et al. 2022). A selected group of intermediate/poor IMDC risk patients may be ideal candidates for nivolumab plus ipilimumab as first-line therapy (Emamekhoo et al. 2022). No evidence is available for the combination of ICIs and TKIs in this scenario. In a cohort study of patients with RCC and brain metastasis, cabozantinib was shown to provide a 55% intracranial response rate for those with progressing disease without local treatment and a 47% intracranial response rate for those with stable disease or progression associated with local therapy (Hirsch et al. 2021). TKIs are considered safe for patients with RCC and brain metastasis, and intracranial bleeding is rare (Hirsch et al. 2021; Unnithan et al. 2007).

**Table:** 

Brain metastasis
Stereotactic body radiation therapy should be used in patients with limited brain metastasis (1–3 lesions)
Whole-brain radiotherapy should be used in patients with several brain metastasis
A combination therapy of ICIs should be used in patients with limited brain metastasis (1–3 lesions), with no/low dose steroids needed

### Osteoclast inhibitors

In metastatic RCC, 20–35% of patients during disease progression develop bone metastases (Wood and Brown 2012), which are highly osteolytic and especially destructive (Nasser et al. 2019). Bone metastases are associated with increased morbidity and decreased quality of life (Wong and Kapoor 2020) as they can lead to several complications, such as spinal cord compression, pathologic fracture, tumour-related hypercalcaemia, pain, and impaired mobility (Chen et al. 2020). Therefore, bone-targeted therapies play a significant role in preventing skeletal-related events (SREs) secondary to metastases.

Zoledronic acid and denosumab are the two main agents used for bone-targeted therapy in patients with metastatic RCC. Among these agents, denosumab has better results in delaying the first SRE in patients with solid tumours, including renal cancer. However, the overall progression of the disease and survival did not significantly differ (Lorange et al. 2023).

Therefore, if there is no contraindication to any agent for metastatic bone disease, the panel has no preference for denosumab or zoledronic acid (recommendation; LE: 1a; GR: B) (Lorange et al. 2023).

The panel recommends the use of 120 mg denosumab every 4 weeks (consensus; LE: 2b; GR: B) (Wong and Kapoor 2020). When using zoledronic acid, scholars in most studies apply 4 mg intravenously; however, a shorter dosage interval (3–4 weeks versus 3 months) was associated with an increased risk of renal impairment, and studies about bone metastases in breast cancer patients did not show a difference between monthly application and application every three months (Polascik and Mouraviev 2008). Thus, the panel recommends the administration of 4 mg of zoledronic acid every 12 weeks (consensus; LE: 5; GR: D).

**Table:** 

Osteoclast inhibitors
There is no preference between denosumab and zoledronic acid for metastatic bone disease if no contraindication to any agent is present
Denosumab should be used in a dose of 120 mg every 4 weeks
Zoledronic acid should be used in a dose of 4 mg every 12 weeks

### Final considerations

Even though targeted therapy and immunotherapy have led to substantial advances in treating advanced RCC, this condition continues to present a challenge and has high mortality rates. Medical consensus requires constant updates as new data become available. In this paper, the LACOG and LARCG present the updated version of the consensus for advanced RCC management in Brazil—especially in the metastatic setting concerning first-line therapy and in the perioperative setting—that could be used in other low- to middle-income countries on the basis of the best evidence in the literature identified and the clinical expertise of our panel.

## Supplementary Information

Below is the link to the electronic supplementary material.

Supplementary file1 (DOCX 48 KB)

## Data Availability

No datasets were generated or analysed during the current study.

## References

[CR1] Abreu D, Carvalhal G, Gueglio G et al (2021) Prognostic factors in de novo metastatic renal cell carcinoma: a report from the Latin American renal cancer group. JCO Glob Oncol 7:671–685 33974442 10.1200/GO.20.00621 PMC8162501

[CR2] Albiges L, Gurney H, Atduev V et al (2023a) Pembrolizumab plus lenvatinib as first-line therapy for advanced non-clear-cell renal cell carcinoma (KEYNOTE-B61): a single-arm, multicentre, phase 2 trial. Lancet Oncol 11:881 10.1016/S1470-2045(23)00276-0 37451291

[CR3] Albiges L, Powles T, Sharma A et al (2023b) CaboPoint: Interim results from a phase 2 study of cabozantinib after checkpoint inhibitor (CPI) therapy in patients with advanced renal cell carcinoma (RCC). J Clin Oncol 41(6):606

[CR4] Alhalabi O, Wilson N, Ajufo H et al (2022) 2022) Safety and differential clinical activity of nivolumab plus ipilimumab (nivo-ipi) in patients (pts) with non-clear cell renal cell carcinoma (nccRCC. J Clin Oncol 40:35634855460

[CR5] Allaf M, Kim SE, Harshman LC et al (2022) Phase III randomized study comparing perioperative nivolumab (nivo) versus observation in patients (Pts) with renal cell carcinoma (RCC) undergoing nephrectomy (PROSPER, ECOG-ACRIN EA8143), a National Clinical Trials Network trial. Ann Oncol 33:S808–S869

[CR6] Amato R, Stepankiw M (2013) Evaluation of everolimus in renal cell cancer. Expert Opin Pharmacother 14(9):1229–1240 23578333 10.1517/14656566.2013.791677

[CR7] Amzal B, Fu S, Meng J, Lister J, Karcher H (2017) Cabozantinib versus everolimus, nivolumab, axitinib, sorafenib and best supportive care: A network meta-analysis of progression-free survival and overall survival in second line treatment of advanced renal cell carcinoma. PLoS ONE 8:0184423 10.1371/journal.pone.0184423 PMC5590935 28886175

[CR8] Auvray M, Auclin E, Barthelemy P et al (2019) Second-line targeted therapies after nivolumab-ipilimumab failure in metastatic renal cell carcinoma. Eur J Cancer 108:33–40 30616146 10.1016/j.ejca.2018.11.031

[CR9] Bahadoram S, Davoodi M, Hassanzadeh S, Bahadoram M, Barahman M, Mafakher L (2022) Renal cell carcinoma: an overview of the epidemiology, diagnosis, and treatment. G Ital Nefrol 39(3):2022 35819037

[CR10] Barata PC, De Liano AG, Mendiratta P et al (2018) The efficacy of VEGFR TKI therapy after progression on immune combination therapy in metastatic renal cell carcinoma. Br J Cancer 119(2):160–163 29795307 10.1038/s41416-018-0104-z PMC6048048

[CR11] Beckermann KE, Sharma D, Chaturvedi S et al (2017) Renal medullary carcinoma: establishing standards in practice. J Oncol Pract 13(7):414–421 28697319 10.1200/JOP.2017.020909 PMC5508447

[CR12] Bex A, Uzzo R, Karam JA et al (2022) LBA66 - IMmotion010: Efficacy and safety from the phase III study of atezolizumab (atezo) vs placebo (pbo) as adjuvant therapy in patients with renal cell carcinoma (RCC) at increased risk of recurrence after resection. Ann Oncol 33:S808–S869

[CR13] Bimbatti D, Ciccarese C, Fantinel E et al (2018) Predictive role of changes in the tumor burden and international metastatic renal cell carcinoma database consortium class during active surveillance for metastatic renal cell carcinoma. Urol Oncol 36(12):526.e13-526.e18 30301699 10.1016/j.urolonc.2018.08.018

[CR14] Blas L, Roberti J, Petroni J, Reniero L, Cicora F (2019) Renal Medullary Carcinoma: a Report of the Current Literature. Curr Urol Rep 20:4 30656488 10.1007/s11934-019-0865-9

[CR15] Broderic J (2023) Adjuvant pembrolizumab improves overall survival in certain patients with renal cell carcinoma. https://www.urologytimes.com/view/adjuvant-pembrolizumab-improves-overall-survival-in-certain-patients-with-renal-cell-carcinoma. Accessed in 12/12/2023.

[CR16] Burotto M, Powles T, Escudier B et al (2023) Nivolumab plus cabozantinib vs sunitinib for first-line treatment of advanced renal cell carcinoma (aRCC): 3-year follow-up from the phase 3 CheckMate 9ER trial. J Clin Oncol 41:603 10.1200/JCO.23.00901 37159880

[CR17] Caliò A, Segala D, Munari E, Brunelli M, Martignoni G (2019) MiT family translocation renal cell carcinoma: from the early descriptions to the current knowledge. Cancers (basel) 11(8):1110 31382581 10.3390/cancers11081110 PMC6721505

[CR18] Calvo E, Escudier B, Motzer RJ et al (2012) Everolimus in metastatic renal cell carcinoma: Subgroup analysis of patients with 1 or 2 previous vascular endothelial growth factor receptor-tyrosine kinase inhibitor therapies enrolled in the phase III RECORD-1 study. Eur J Cancer 48(3):333–339 22209391 10.1016/j.ejca.2011.11.027

[CR19] Cannady SB, Cavanaugh KA, Lee SY et al (2004) Results of whole brain radiotherapy and recursive partitioning analysis in patients with brain metastases from renal cell carcinoma: a retrospective study. Int J Radiat Oncol Biol Phys 58(1):253–258 14697446 10.1016/s0360-3016(03)00818-6

[CR20] CEBM (2009) Oxford Centre for Evidence-Based Medicine 2009 Levels of Evidence and Grades of Recommendations. https://www.cebm.ox.ac.uk/resources/levels-of-evidence/oxford-centre-for-evidence-based-medicine-levels-of-evidence-march-2009 Accessed on 10 June 2023.

[CR21] Chahoud J, Msaouel P, Campbell MT et al (2020) Nivolumab for the treatment of patients with metastatic non-clear cell renal cell carcinoma (nccRCC): a single-institutional experience and literature meta-analysis. Oncologist 25(3):252–258 32162795 10.1634/theoncologist.2019-0372 PMC7066696

[CR22] Chen J, Zhou L, Liu X, Wen X, Li H, Li W (2020) Meta-analysis of clinical trials to assess denosumab over zoledronic acid in bone metastasis. Int J Clin Pharm 43(1):2–10 32964403 10.1007/s11096-020-01105-1

[CR23] Choueiri TK, Escudier B, Powles T et al (2016) Cabozantinib versus everolimus in advanced renal cell carcinoma (METEOR): final results from a randomised, open-label, phase 3 trial. Lancet Oncol 17(7):917–927 27279544 10.1016/S1470-2045(16)30107-3

[CR24] Choueiri TK, Halabi S, Sanford BL et al (2017) Cabozantinib versus sunitinib as initial targeted therapy for patients with metastatic renal cell carcinoma of poor or intermediate risk: The Alliance A031203 CABOSUN Trial. J Clin Oncol 35(6):591–597 28199818 10.1200/JCO.2016.70.7398 PMC5455807

[CR25] Choueiri TK, Larkin J, Pal S et al (2021a) Efficacy and correlative analyses of avelumab plus axitinib versus sunitinib in sarcomatoid renal cell carcinoma: post hoc analysis of a randomized clinical trial. ESMO 6(3):100101 10.1016/j.esmoop.2021.100101 PMC8099757 33901870

[CR26] Choueiri TK, Powles T, Burotto M et al (2021b) Nivolumab plus cabozantinib versus sunitinib for advanced renal-cell carcinoma. N Engl J Med 384(9):829–841 33657295 10.1056/NEJMoa2026982 PMC8436591

[CR27] Choueiri TK, Powles T, Albiges L et al (2023) Cabozantinib plus Nivolumab and Ipilimumab in renal-cell carcinoma. N Engl J Med 388(19):1767–1778 37163623 10.1056/NEJMoa2212851 PMC10257898

[CR28] Choueiri TK, Tomczak P, Park SH, et al (2021a) KEYNOTE-564 Investigators. Adjuvant Pembrolizumab after Nephrectomy in Renal-Cell Carcinoma. N Engl J Med 385(8):683–694. 10.1056/NEJMoa2106391 34407342

[CR29] Ciccarese C, Iacovelli R, Brunelli M et al (2017) Addressing the best treatment for non-clear cell renal cell carcinoma: A meta-analysis of randomised clinical trials comparing VEGFR-TKis versus mTORi-targeted therapies. Eur J Cancer 83:237–246 28756136 10.1016/j.ejca.2017.06.030

[CR30] Correa AF, Jegede OA, Haas NB et al (2021) Predicting disease recurrence, early progression, and overall survival following surgical resection for high-risk localized and locally advanced renal cell carcinoma. Eur Urol 80(1):20–31 33707112 10.1016/j.eururo.2021.02.025 PMC8627688

[CR31] Dason S, Allard C, Sheridan-Jonah A et al (2013) Management of renal collecting duct carcinoma: a systematic review and the McMaster experience. Curr Oncol 20(3):e223-232 23737692 10.3747/co.20.1230 PMC3671029

[CR32] Eisen T, Frangou E, Oza B et al (2020) Adjuvant sorafenib for renal cell carcinoma at intermediate or high risk of relapse: results from the sorce randomized phase III intergroup trial. J Clin Oncol 38(34):4064–4075 33052759 10.1200/JCO.20.01800 PMC7768344

[CR33] Emamekhoo H, Olsen MR, Carthon BC et al (2022) Safety and efficacy of nivolumab plus ipilimumab in patients with advanced renal cell carcinoma with brain metastases: CheckMate 920. Cancer 128(5):966–974 34784056 10.1002/cncr.34016 PMC9298991

[CR34] Escudier B, Motzer RJ, Dyer M et al (2022) 1459P - Analysis of long-term efficacy outcomes from the CheckMate 025 (CM 025) trial comparing nivolumab (NIVO) vs everolimus (EVE) based on ≥ 7 years (yrs) of follow-up in pre-treated patients (pts) with advanced renal cell carcinoma (aRCC). Ann Oncol 33:S660–S680

[CR35] Fernández-Pello S, Hofmann F, Tahbaz R et al (2017) A systematic review and meta-analysis comparing the effectiveness and adverse effects of different systemic treatments for non-clear cell renal cell carcinoma. Eur Urol 71(3):426–436 27939075 10.1016/j.eururo.2016.11.020

[CR36] Fyfe G, Fisher RI, Rosenberg SA et al (1995) Results of treatment of 255 patients with metastatic renal cell carcinoma who received high-dose recombinant interleukin-2 therapy. J Clin Oncol 13:688 7884429 10.1200/JCO.1995.13.3.688

[CR37] Gross-Goupil M, Kwon TG, Eto M et al (2018) Axitinib versus placebo as an adjuvant treatment of renal cell carcinoma: results from the phase III, randomized ATLAS trial. Ann Oncol 29(12):2371–2378 30346481 10.1093/annonc/mdy454 PMC6311952

[CR38] Gupta R, Ornstein MC, Li H et al (2020) Clinical activity of Ipilimumab plus Nivolumab in patients with metastatic non-clear cell renal cell carcinoma. Clin Genitourin Cancer 18(6):429–435 32800717 10.1016/j.clgc.2019.11.012

[CR39] Haanen JBAG, Larkin J, Choueiri TK et al (2023) Extended follow-up from JAVELIN Renal 101: subgroup analysis of avelumab plus axitinib versus sunitinib by the International Metastatic Renal Cell Carcinoma Database Consortium risk group in patients with advanced renal cell carcinoma. ESMO Open 8(3):101210 37104931 10.1016/j.esmoop.2023.101210 PMC10265611

[CR40] Haas NB, Manola J, Uzzo RG et al (2016) Adjuvant sunitinib or sorafenib for high-risk, non-metastatic renal-cell carcinoma (ECOG-ACRIN E2805): a double-blind, placebo-controlled, randomised, phase 3 trial. Lancet 387(10032):2008–2016 26969090 10.1016/S0140-6736(16)00559-6 PMC4878938

[CR41] Haas NB, Manola J, Dutcher JP et al (2017) Adjuvant treatment for high-risk clear cell renal cancer: updated results of a high-risk subset of the ASSURE randomized trial. JAMA Oncol 3(9):1249–1252 28278333 10.1001/jamaoncol.2017.0076 PMC5591751

[CR42] Hanif A, Pandey M, Khan S, Attwood K, George S (2019) Metastatic sarcomatoid renal cell carcinoma treated with immune checkpoint inhibitors. Oncoimmunology 8(8):1606639 31413914 10.1080/2162402X.2019.1606639 PMC6682356

[CR43] Hansen HC, Janssen S, Schild SE, Rades D (2019) Estimating survival of patients with metastatic renal cell carcinoma receiving whole-brain radiotherapy with a new tool. Anticancer Res 39(4):2091–2095 30952754 10.21873/anticanres.13321

[CR44] Harrison MR, Costello BA, Bhavsar NA et al (2021) Active surveillance of metastatic renal cell carcinoma: Results from a prospective observational study (MaRCC). Cancer 127(13):2204–2212 33765337 10.1002/cncr.33494 PMC8251950

[CR45] Hasanov E, Yeboa DN, Tucker MD et al (2022) An interdisciplinary consensus on the management of brain metastases in patients with renal cell carcinoma. CA Cancer J Clin 72(5):454–489 35708940 10.3322/caac.21729

[CR46] Heng DY, Xie W, Regan MM et al (2009) Prognostic factors for overall survival in patients with metastatic renal cell carcinoma treated with vascular endothelial growth factor-targeted agents: Results from a large, multicenter study. J Clin Oncol 27:5794–5799 19826129 10.1200/JCO.2008.21.4809

[CR47] Heng DY, Xie W, Regan MM et al (2013) External validation and comparison with other models of the international metastatic renal-cell carcinoma database consortium prognostic model: a population-based study. Lancet Oncol 14(2):141–148 23312463 10.1016/S1470-2045(12)70559-4 PMC4144042

[CR48] Heo JH, Park C, Ghosh S et al (2021) A network meta-analysis of efficacy and safety of first-line and second-line therapies for the management of metastatic renal cell carcinoma. J Clin Pharm Ther 46(1):35–49 33112003 10.1111/jcpt.13282

[CR49] Hirsch L, Martinez Chanza N, Farah S et al (2021) Clinical activity and safety of cabozantinib for brain metastases in patients with renal cell carcinoma. JAMA Oncol 7(12):1815–1823 34673916 10.1001/jamaoncol.2021.4544 PMC8532040

[CR50] Hutson TE, Michaelson MD, Kuzel TM et al (2021) A single-arm, multicenter, phase 2 study of Lenvatinib plus everolimus in patients with advanced non-clear cell renal cell carcinoma. Eur Urol 80(2):162–170 33867192 10.1016/j.eururo.2021.03.015 PMC12684810

[CR51] Iacovelli R, Modica D, Palazzo A et al (2015) Clinical outcome and prognostic factors in renal medullary carcinoma: A pooled analysis from 18 years of medical literature. Can Urol Assoc J 9(3–4):E172–E177 26085875 10.5489/cuaj.2373 PMC4455635

[CR52] Ippen FM, Mahadevan A, Wong ET et al (2015) Stereotactic radiosurgery for renal cancer brain metastasis: prognostic factors and the role of whole-brain radiation and surgical resection. J Oncol 2015:636918 26681942 10.1155/2015/636918 PMC4668321

[CR53] Ishihara H, Takagi T, Kondo T et al (2018) Efficacy and safety of third-line molecular-targeted therapy in metastatic renal cell carcinoma resistant to first-line vascular endothelial growth factor receptor tyrosine kinase inhibitor and second-line therapy. Int J Clin Oncol 23(3):559–567 29327159 10.1007/s10147-018-1241-3

[CR54] Jessurun CAC, Hulsbergen AFC, de Wit AE et al (2021) The combined use of steroids and immune checkpoint inhibitors in brain metastasis patients: a systematic review and meta-analysis. Neuro Oncol 23(8):1261–1272 33631792 10.1093/neuonc/noab046 PMC8328028

[CR55] Jung KS, Lee SJ, Park SH et al (2018) Pazopanib for the treatment of non-clear cell renal cell carcinoma: a single-arm, open-label, multicenter Phase II Study. Cancer Res Treat 50(2):488–494 28546525 10.4143/crt.2016.584 PMC5912146

[CR56] Karner C, Kew K, Wakefield V, Masento N, Edwards SJ (2019) Targeted therapies for previously treated advanced or metastatic renal cell carcinoma: systematic review and network meta-analysis. BMJ Open 9(3):024691 10.1136/bmjopen-2018-024691 PMC6429896 30826762

[CR57] Kidney cancer statistics. https://www.wcrf.org/cancer-trends/kidney-cancer-statistics/ Accessed on 10 June 2023.

[CR58] Kilari D, Szabo A, Ghatalia P et al (2021) Outcomes with novel combinations in non-clear cell renal cell carcinoma(nccRCC): ORACLE study. J Clin Oncol 39:4580

[CR59] Knox JJ, Barrios CH, Kim TM et al (2017) Final overall survival analysis for the phase II RECORD-3 study of first-line everolimus followed by sunitinib versus first-line sunitinib followed by everolimus in metastatic RCC. Ann Oncol 28(6):1339–1345 28327953 10.1093/annonc/mdx075 PMC5452072

[CR60] Kushnir I, Basappa NS, Ghosh S et al (2021) Active Surveillance in Metastatic Renal Cell Carcinoma: Results From the Canadian Kidney Cancer Information System. Clin Genitourin Cancer 19(6):521–530 34158246 10.1016/j.clgc.2021.05.004

[CR61] Lee CH, Shah AY, Rasco D et al (2021a) Lenvatinib plus pembrolizumab in patients with either treatment-naive or previously treated metastatic renal cell carcinoma (Study 111/KEYNOTE-146): a phase 1b/2 study. Lancet Oncol 22(7):946–958 34143969 10.1016/S1470-2045(21)00241-2 PMC8316679

[CR62] Lee JL, McDermott DF, Ziobro M et al (2021b) Pembrolizumab (pembro) monotherapy as first-line therapy in advanced noneclear cell renal cell carcinoma (nccRCC): Results after a minimum of 34 months of follow-up from KEYNOTE-427 cohort B. Ann Oncol 32(5):S687

[CR63] Lee CH, Fitzgerald KN, Voss MH et al (2023) Nivolumab plus cabozantinib in patients with non-clear cell renal cell carcinoma: Updated results from a phase 2 trial. J Clin Oncol 41:4537 10.1016/j.eururo.2024.04.025 PMC11970537 38782695

[CR64] Leow JJ, Chong YL, Chang SL et al (2021) Neoadjuvant and adjuvant chemotherapy for upper tract urothelial carcinoma: a 2020 systematic review and meta-analysis, and future perspectives on systemic therapy. Eur Urol 79(5):635–654 32798146 10.1016/j.eururo.2020.07.003

[CR65] Levitin M, Ofori J, Shin WJ et al (2020) Radiation and checkpoint inhibitor immunotherapy lead to long term disease control in a metastatic RCC patient with brain metastases. Front Oncol 10:566070 33072598 10.3389/fonc.2020.566070 PMC7539115

[CR66] Lindner AK, Tulchiner G, Seeber A et al (2022) Targeting strategies in the treatment of fumarate hydratase deficient renal cell carcinoma. Front Oncol 12:906014 35912170 10.3389/fonc.2022.906014 PMC9337267

[CR67] Lorange JP, Ramirez Garcia Luna J, Grou-Boileau F et al (2023) Management of bone metastasis with zoledronic acid: A systematic review and Bayesian network meta-analysis. J Bone Oncol 39:100470 36860585 10.1016/j.jbo.2023.100470 PMC9969300

[CR68] Lu Y, Song Y, Xu Y et al (2020) The prevalence and prognostic and clinicopathological value of PD-L1 and PD-L2 in renal cell carcinoma patients: a systematic review and meta-analysis involving 3,389 patients. Transl Androl Urol 9(2):367–381 32420142 10.21037/tau.2020.01.21 PMC7215048

[CR69] Luke JJ, Robert C, Carlino MS et al (2022) Safety profile of adjuvant pembrolizumab (pembro) in melanoma, non-small cell lung cancer (NSCLC), and renal cell carcinoma (RCC): Pooled analysis of phase III clinical trials. Ann Oncol 16:100102

[CR70] Matsui Y (2020) Current multimodality treatments against brain metastases from renal cell carcinoma. Cancers (basel) 12(10):2875 33036276 10.3390/cancers12102875 PMC7600559

[CR71] Meyer E, Pasquier D, Bernadou G et al (2018) Stereotactic radiation therapy in the strategy of treatment of metastatic renal cell carcinoma: A study of the Getug group. Eur J Cancer 98:38–47 29864737 10.1016/j.ejca.2018.04.008

[CR72] Monteiro FSM, Soares A, Rizzo A, Santoni M et al (2023) The role of immune checkpoint inhibitors (ICI) as adjuvant treatment in renal cell carcinoma (RCC): A systematic review and meta-analysis. Clin Genitourin Cancer 21(3):324–333 36823017 10.1016/j.clgc.2023.01.005

[CR73] Motzer RJ, Escudier B, Oudard S et al (2008) Efficacy of everolimus in advanced renal cell carcinoma: a double-blind, randomised, placebo-controlled phase III trial. Lancet 372(9637):449–456 18653228 10.1016/S0140-6736(08)61039-9

[CR74] Motzer RJ, Hutson TE, Cella D et al (2013) Pazopanib versus sunitinib in metastatic renal-cell carcinoma. N Engl J Med 369(8):722–731 23964934 10.1056/NEJMoa1303989

[CR75] Motzer RJ, Escudier B, McDermott DF et al (2015a) Nivolumab versus everolimus in advanced renal-cell carcinoma. N Engl J Med 373(19):1803–1813 26406148 10.1056/NEJMoa1510665 PMC5719487

[CR76] Motzer RJ, Hutson TE, Glen H et al (2015b) Lenvatinib, everolimus, and the combination in patients with metastatic renal cell carcinoma: a randomised, phase 2, open-label, multicentre trial. Lancet Oncol 16(15):1473–1482 26482279 10.1016/S1470-2045(15)00290-9

[CR77] Motzer RJ, Tannir NM, McDermott DF et al (2018) Nivolumab plus Ipilimumab versus Sunitinib in advanced renal-cell carcinoma. N Engl J Med 378(14):1277–1290 29562145 10.1056/NEJMoa1712126 PMC5972549

[CR78] Motzer RJ, Penkov K, Haanen J et al (2019) Avelumab plus Axitinib versus Sunitinib for advanced renal-cell carcinoma. N Engl J Med 380(12):1103–1115 30779531 10.1056/NEJMoa1816047 PMC6716603

[CR79] Motzer RJ, Escudier B, George S et al (2020) Nivolumab versus everolimus in patients with advanced renal cell carcinoma: Updated results with long-term follow-up of the randomized, open-label, phase 3 CheckMate 025 trial. Cancer 126(18):4156–4167 32673417 10.1002/cncr.33033 PMC8415096

[CR80] Motzer R, Alekseev B, Rha SY et al (2021a) Lenvatinib plus pembrolizumab or everolimus for advanced renal cell carcinoma. N Engl J Med 384(14):1289–1300 33616314 10.1056/NEJMoa2035716

[CR81] Motzer RJ, Choueiri TK, Powles T et al (2021b) Nivolumab + cabozantinib (NIVO+CABO) versus sunitinib (SUN) for advanced renal cell carcinoma (aRCC): Outcomes by sarcomatoid histology and updated trial results with extended follow-up of CheckMate 9ER. J Clin Oncol 39:30833356420

[CR82] Motzer RJ, Russo P, Haas N et al (2021c) Adjuvant pazopanib versus placebo after nephrectomy in patients with localized or locally advanced renal cell carcinoma: final overall survival analysis of the phase 3 PROTECT trial. Eur Urol 79(3):334–338 33461782 10.1016/j.eururo.2020.12.029 PMC8407394

[CR83] Motzer RJ, McDermott DF, Escudier B et al (2022a) Conditional survival and long-term efficacy with nivolumab plus ipilimumab versus sunitinib in patients with advanced renal cell carcinoma. Cancer 128(11):2085–2097 35383908 10.1002/cncr.34180 PMC9543316

[CR84] Motzer RJ, Powles T, Burotto M et al (2022b) Nivolumab plus cabozantinib versus sunitinib in first-line treatment for advanced renal cell carcinoma (CheckMate 9ER): long-term follow-up results from an open-label, randomised, phase 3 trial. Lancet Oncol 23(7):888–898 35688173 10.1016/S1470-2045(22)00290-X PMC10305087

[CR85] Motzer RJ, Porta C, Eto M et al (2023a) Final prespecified overall survival (OS) analysis of CLEAR: 4-year follow-up of lenvatinib plus pembrolizumab (L+P) vs sunitinib (S) in patients (pts) with advanced renal cell carcinoma (aRCC). J Clin Oncol 41:4502–4502

[CR86] Motzer RJ, Russo P, Grünwald V et al (2023b) Adjuvant nivolumab plus ipilimumab versus placebo for localised renal cell carcinoma after nephrectomy (CheckMate 914): a double-blind, randomised, phase 3 trial. Lancet 401(10379):821–832 36774933 10.1016/S0140-6736(22)02574-0 PMC10259621

[CR87] Mourão TC, Curado MP, de Oliveira RAR et al (2022) Epidemiology of urological cancers in brazil: trends in mortality rates over more than two decades. J Epidemiol Glob Health 12(3):239–247 35639266 10.1007/s44197-022-00042-8 PMC9470798

[CR88] Nabors LB, Portnow J, Ammirati M et al (2014) Central nervous system cancers, version 2. 2014. Featured updates to the NCCN Guidelines. J Natl Compr Cancer Netw 12:1517–1523 10.6004/jnccn.2014.0151 PMC4337873 25361798

[CR89] Nasser SM, Sahal A, Hamad A, Elazzazy S (2019) Effect of denosumab versus zoledronic acid on calcium levels in cancer patients with bone metastasis: A retrospective cohort study. J Oncol Pharm Pract 25(8):1846–1852 31694497 10.1177/1078155218820927 PMC6838728

[CR90] Nieder C, Spanne O, Mehta MP, Grosu AL, Geinitz H (2011) Presentation, patterns of care, and survival in patients with brain metastases: what has changed in the last 20 years? Cancer 117(11):2505–2512 24048799 10.1002/cncr.25707

[CR91] Nizam A, Schindelheim JA, Ornstein MC (2020) The role of active surveillance and cytoreductive nephrectomy in metastatic renal cell carcinoma. Cancer Treat Res Commun 23:100169 32126518 10.1016/j.ctarc.2020.100169

[CR92] Osterman CK, Rose TL (2020) A systematic review of systemic treatment options for advanced non-clear cell renal cell carcinoma. Kidney Cancer 4(1):15–27 34435168 10.3233/kca-190078 PMC8384265

[CR93] Padala SA, Barsouk A, Thandra KC et al (2020) Epidemiology of renal cell carcinoma. World J Oncol 11(3):79–87 32494314 10.14740/wjon1279 PMC7239575

[CR94] Pal SK, Tangen C, Thompson IM Jr et al (2021) A comparison of sunitinib with cabozantinib, crizotinib, and savolitinib for treatment of advanced papillary renal cell carcinoma: a randomised, open-label, phase 2 trial. Lancet 397(10275):695–703 33592176 10.1016/S0140-6736(21)00152-5 PMC8687736

[CR95] Pal SK, Albiges L, Tomczak P et al (2023) Atezolizumab plus cabozantinib versus cabozantinib monotherapy for patients with renal cell carcinoma after progression with previous immune checkpoint inhibitor treatment (CONTACT-03): a multicentre, randomised, open-label, phase 3 trial. Lancet 402(10397):185–195 37290461 10.1016/S0140-6736(23)00922-4 PMC11017728

[CR96] Park I, Lee JL, Ahn JH et al (2014) Active surveillance for metastatic or recurrent renal cell carcinoma. J Cancer Res Clin Oncol 140(8):1421–1428 24752340 10.1007/s00432-014-1680-9 PMC11823793

[CR97] Polascik TJ, Mouraviev V (2008) Zoledronic acid in the management of metastatic bone disease. Ther Clin Risk Manag 4(1):261–268 18728715 10.2147/tcrm.s2707 PMC2503661

[CR98] Powles T, Tomczak P, Park SH et al (2022) Pembrolizumab versus placebo as post-nephrectomy adjuvant therapy for clear cell renal cell carcinoma (KEYNOTE-564): 30-month follow-up analysis of a multicentre, randomised, double-blind, placebo-controlled, phase 3 trial. Lancet Oncol 23(9):1133–1144 36055304 10.1016/S1470-2045(22)00487-9

[CR99] Rassy E, Flippot R, Albiges L (2020) Tyrosine kinase inhibitors and immunotherapy combinations in renal cell carcinoma. Ther Adv Med Oncol 12:1758835920907504 32215057 10.1177/1758835920907504 PMC7081462

[CR100] Ravaud A, Motzer RJ, Pandha HS et al (2016) Adjuvant sunitinib in high-risk renal-cell carcinoma after nephrectomy. N Engl J Med 375(23):2246–2254 27718781 10.1056/NEJMoa1611406

[CR101] Raychaudhuri R, Riese MJ, Bylow K et al (2017) Immune check point inhibition in sarcomatoid renal cell carcinoma: a new treatment paradigm. Clin Genitourin Cancer 15(5):e897–e901 28645484 10.1016/j.clgc.2017.05.018

[CR102] Rini BI, Dorff TB, Elson P et al (2016) Active surveillance in metastatic renal-cell carcinoma: a prospective, phase 2 trial. Lancet Oncol 17(9):1317–1324 27498080 10.1016/S1470-2045(16)30196-6 PMC9199453

[CR103] Rini BI, Plimack ER, Stus V et al (2019a) Pembrolizumab (pembro) plus axitinib (axi) versus sunitinib as first-line therapy for metastatic renal cell carcinoma (mRCC): Outcomes in the combined IMDC intermediate/poor risk and sarcomatoid subgroups of the phase 3 KEYNOTE-426 study. J Clin Oncol 37:4500

[CR104] Rini BI, Plimack ER, Stus V et al (2019b) Pembrolizumab plus Axitinib versus Sunitinib for advanced renal-cell carcinoma. N Engl J Med 380(12):1116–1127 30779529 10.1056/NEJMoa1816714

[CR105] Rini BI, Plimack ER, Stus V et al (2023) Pembrolizumab plus axitinib versus sunitinib as first-line therapy for advanced clear cell renal cell carcinoma: 5-year analysis of KEYNOTE-426. J Clin Oncol 41:LBA4501

[CR106] Ryan CW, Tangen C, Heath EI et al (2022) EVEREST: Everolimus for renal cancer ensuing surgical therapy—A phase III study (SWOG S0931, NCT01120249). J Clin Oncol 40:LBA4500

[CR107] Sameh WM, Hashad MM, Eid AA, Abou Yousif TA, Atta MA (2012) Recurrence pattern in patients with locally advanced renal cell carcinoma: The implications of clinicopathological variables. Arab J Urol 10(2):131–137 26558015 10.1016/j.aju.2011.12.007 PMC4442897

[CR108] Santoni M, Aurilio G, Massari F et al (2022) Nivolumab versus cabozantinib as second-line therapy in patients with advanced renal cell carcinoma: a real-world comparison. Clin Genitourin Cancer 20(3):285–295 35305916 10.1016/j.clgc.2022.02.003

[CR109] Santoni M, Massari F, Myint ZW et al (2023) Global real-world outcomes of patients receiving immuno-oncology combinations for advanced renal cell carcinoma: the ARON-1 study. Target Oncol 18(4):559–570 37369815 10.1007/s11523-023-00978-2

[CR110] Shah AY, Kotecha RR, Lemke EA et al (2019) Outcomes of patients with metastatic clear-cell renal cell carcinoma treated with second-line VEGFR-TKI after first-line immune checkpoint inhibitors. Eur J Cancer 114:67–75 31075726 10.1016/j.ejca.2019.04.003 PMC7537491

[CR111] Sharma T, Tajzler C, Kapoor A (2018) Is there a role for adjuvant therapy after surgery in “High Risk for Recurrence” Kidney Cancer? an update on current concepts. Curr Oncol 25(5):444–453 10.3747/co.25.3865 PMC6209555 30464696

[CR112] Siegel RL, Miller KD, Wagle NS (2023) Cancer statistics, 2023. CA Cancer J Clin 73(1):17–48 36633525 10.3322/caac.21763

[CR113] Sneed GT, Lee S, Brown JN, Hammond JM (2019) The role of pazopanib in non-clear cell renal cell carcinoma: a systematic review. Clin Genitourin Cancer 17(6):419–424 31585694 10.1016/j.clgc.2019.09.001

[CR114] Soares A, Monteiro FSM, Maluf FC et al (2020) Advanced renal cell carcinoma (RCC) management: an expert panel recommendation from the Latin American Cooperative Oncology Group (LACOG) and the Latin American Renal Cancer Group (LARCG). J Cancer Res Clin Oncol 146(7):1829–1845 32410064 10.1007/s00432-020-03236-4 PMC7256074

[CR115] Srinivasan R, Gurram S, Al Harthy M et al (2020) Results from a phase II study of bevacizumab and erlotinib in subjects with advanced hereditary leiomyomatosis and renal cell cancer (HLRCC) or sporadic papillary renal cell cancer. JCO 38:5004

[CR116] Sun M, De Velasco G, Brastianos PK et al (2019) The development of brain metastases in patients with renal cell carcinoma: epidemiologic trends, survival, and clinical risk factors using a population-based cohort. Eur Urol Focus 5(3):474–481 29311016 10.1016/j.euf.2017.12.007

[CR117] Tannir NM, Plimack E, Ng C et al (2012) A phase 2 trial of sunitinib in patients with advanced non-clear cell renal cell carcinoma. Eur Urol 62(6):1013–1019 22771265 10.1016/j.eururo.2012.06.043 PMC3882163

[CR118] Tannir NM, Jonasch E, Albiges L et al (2016) Everolimus versus sunitinib prospective evaluation in metastatic non-clear cell renal cell carcinoma (ESPN): a randomized multicenter phase 2 trial. Eur Urol 69(5):866–874 26626617 10.1016/j.eururo.2015.10.049 PMC4879109

[CR119] Tannir NM, Signoretti S, Choueiri TK et al (2021) Efficacy and safety of nivolumab plus ipilimumab versus sunitinib in first-line treatment of patients with advanced sarcomatoid renal cell carcinoma. Clin Cancer Res 27(1):78–86 32873572 10.1158/1078-0432.CCR-20-2063 PMC8589223

[CR120] Tenold M, Ravi P, Kumar M et al (2020) Current approaches to the treatment of advanced or metastatic renal cell carcinoma. Am Soc Clin Oncol Educ Book 40:1–10 32239988 10.1200/EDBK_279881

[CR121] Thomas AA, Rini BI, Lane BR, Garcia J, Dreicer R, Klein EA, Novick AC, Campbell SC (2009) Response of the primary tumor to neoadjuvant sunitinib in patients with advanced renal cell carcinoma. J Urol 181(2):518–523 19100579 10.1016/j.juro.2008.10.001

[CR122] Tran J, Ornstein MC (2022) Clinical review on the management of metastatic renal cell carcinoma. JCO Oncol Pract 18(3):187–196 34529499 10.1200/OP.21.00419

[CR123] Tykodi SS, Gordan LN, Alter RS et al (2022) Safety and efficacy of nivolumab plus ipilimumab in patients with advanced non-clear cell renal cell carcinoma: results from the phase 3b/4 CheckMate 920 trial. J Immunother Cancer 10(2):e003844 35210307 10.1136/jitc-2021-003844 PMC8883262

[CR124] Unnithan JS, Choueiri T, Garcia J et al (2007) Safety of VEGF-targeted tyrosine kinase inhibitors in patients (Pts) with metastatic renal cell carcinoma (mRCC) and central nervous system (CNS) metastases. J Clin Oncol 25:504717971612

[CR125] Vera-Badillo FE, Templeton AJ, Duran I et al (2015) Systemic therapy for non-clear cell renal cell carcinomas: a systematic review and meta-analysis. Eur Urol 67(4):740–749 24882670 10.1016/j.eururo.2014.05.010

[CR126] Wei Q, He H, Lv L, Xu X, Sun W (2020) The promising role of radiotherapy in the treatment of advanced or metastatic renal cell carcinoma: a narrative review. Transl Androl Urol 9(6):2821–2830 33457253 10.21037/tau-20-1466 PMC7807309

[CR127] WHO. Kidney cancer fact sheet. https://gco.iarc.fr/today/data/factsheets/cancers/29-Kidney-fact-sheet.pdf Accessed on 10 June 2023.

[CR128] Wiecek W, Karcher H (2016) Nivolumab versus cabozantinib: comparing overall survival in metastatic renal cell carcinoma. PLoS ONE 11(6):e0155389 27271250 10.1371/journal.pone.0155389 PMC4894561

[CR129] Wong ECL, Kapoor A (2020) Does bone-targeted therapy benefit patients with metastatic renal cell carcinoma? Transl Oncol 13(2):241–244 31869748 10.1016/j.tranon.2019.10.009 PMC6931200

[CR130] Wood SL, Brown JE (2012) Skeletal metastasis in renal cell carcinoma: current and future management options. Cancer Treat Rev 38(4):284–291 21802857 10.1016/j.ctrv.2011.06.011

[CR131] Yang J, Wang K, Yang Z (2023) Treatment strategies for clear cell renal cell carcinoma: past, present and future. Front Oncol 13:1133832 37025584 10.3389/fonc.2023.1133832 PMC10070676

