# Supplementary material for: Advanced renal cell carcinoma management: the Latin American Cooperative Oncology Group (LACOG) and the Latin American Renal Cancer Group (LARCG) consensus update
Source: J Cancer Res Clin Oncol. 2024 Apr 9;150(4):183. doi: 10.1007/s00432-024-05663-z (PMC11003910; doi:10.1007/s00432-024-05663-z)
Supplement: Supplementary file 1 — Supplementary file1 (DOCX 48 KB) [file 432_2024_5663_MOESM1_ESM.docx]

**Supplementary file**

**Adjuvant treatment**

**1) Do you recommend adjuvant treatment (pembrolizumab) in patients with clear cell carcinoma pT2N0 and grade 4 Fuhrman and/or sarcomatoid features?**

a) Yes, in a majority of patients 33.82%

b) Yes, in a minority of patients 42.65%

c) No 23.53%

d) Abstain

**1-revoted) Do you recommend adjuvant treatment (pembrolizumab) in patients with clear cell carcinoma pT2N0 and grade 4 Fuhrman and/or sarcomatoid features?**

a) Yes, in a majority of patients 19%

**b) Yes, in a minority of patients 51%**

c) No 30%

d) Abstain

**2) Do you recommend adjuvant treatment (sunitinib or pembrolizumab) in patients with clear cell carcinoma > pT3N0 any grade?**

a) Yes, in a majority of patients 34.78%

b) Yes, in a minority of patients 42.03%

c) No 23.19%

d) Abstain

**2-revoted) Do you recommend adjuvant treatment (sunitinib or pembrolizumab) in patients with clear cell carcinoma > pT3N0 any grade?**

a) Yes, in a majority of patients 18%

**b) Yes, in a minority of patients 59%**

c) No 23%

d) Abstain

**3) When you recommend adjuvant treatment in patients with clear cell carcinoma > pT3N0 any grade, do you recommend?**

a) Yes, in a majority of patients 4.62%

**b) Yes, in a minority of patients 95.38%**

c) No 0%

d) Abstain

**4) Do you recommend adjuvant treatment in patients with clear cell carcinoma pTanyN+ any grade?**

a) Yes, in a majority of patients 67.16%

b) Yes, in a minority of patients 26.87%

c) No 5.97%

d) Abstain

**4-revoted) Do you recommend adjuvant treatment in patients with clear cell carcinoma pTanyN+ any grade?**

**a) Yes, in a majority of patients 56%**

b) Yes, in a minority of patients 42%

c) No 2%

d) Abstain

**5) When you recommend adjuvant treatment in patients with clear cell carcinoma pTanyN+ any grade, do you recommend?**

a) Sunitinib 4.69%

**b) Pembrolizumab 95.31%**

c) Abstain

**6) Do you recommend adjuvant treatment (pembrolizumab) in patients with clear cell carcinoma pTanyNanyM1 NED any grade?**

a) Yes, in a majority of patients 67.69%

b) Yes, in a minority of patients 24.62%

c) No 7.69%

d) Abstain

**6-revoted) Do you recommend adjuvant treatment (pembrolizumab) in patients with clear cell carcinoma pTanyNanyM1 NED any grade?**

**a) Yes, in a majority of patients 51%**

b) Yes, in a minority of patients 37%

c) No 12%

d) Abstain

**7) Do you recommend adjuvant treatment (sunitinib) in patients with non-clear cell carcinoma > pT3N0 any grade?**

a) Yes, in a majority of patients 4.69%

b) Yes, in a minority of patients 12.50%

**c) No 82.81%**

d) Abstain

**8) When you recommend sunitinib as adjuvant therapy, do you start with a full dose?**

**a) Yes 76.92%**

b) No 23.08%

c) Abstain

**9) If dose reduction is necessary (sunitinib), do you discontinue the treatment?**

a) Yes 29.17%

b) No 70.83%

c) Abstain

**9-revoted) If dose reduction is necessary (sunitinib), do you discontinue the treatment?**

**a) Yes 82%**

b) No 18%

c) Abstain

**10) When do you adjust the dose (sunitinib)?**

a) Occurrence of any grade III toxicity 52%

b) Occurrence of any grade II toxicity 48%

c) Abstain

**10-revoted) When do you adjust the dose (sunitinib)?**

a) Occurrence of any grade III toxicity 33%

**b) Occurrence of any grade II toxicity 67%**

c) Abstain

**11) When do you interrupt the treatment (sunitinib)?**

**a) Occurrence of any grade III toxicity 96.30%**

b) Occurrence of any grade II toxicity 3.70%

c) Abstain

**Neoadjuvant treatment**

**12) Do you recommend neoadjuvant treatment in patients with clear cell carcinoma?**

a) Yes, in a majority of patients 0%

b) Yes, in a very selected cases, after a multidisciplinary team discussion 73.24%

c) No 26.76%

d) Abstain

**12-revoted) Do you recommend neoadjuvant treatment in patients with clear cell carcinoma?**

a) Yes, in a majority of patients 0%

**b) Yes, in a very selected cases, after a multidisciplinary team discussion 100%**

c) No 0%

d) Abstain

**13) Do you recommend neoadjuvant treatment in patients with both kidneys and resectable locally advanced disease?**

a) Yes 11.43%

**b) No 88.57%**

c) Abstain

**14) Do you recommend neoadjuvant treatment in patients with both kidneys and irresectable locally advanced disease?**

**a) Yes 81.16%**

b) No 18.84%

c) Abstain

**15) Do you recommend neoadjuvant treatment in patients with solitary kidney and candidate to partial nephrectomy?**

a) Yes 21.43%

**b) No 78.57%**

c) Abstain

**16) Do you recommend neoadjuvant treatment in patients with solitary kidney and NOT candidate to partial nephrectomy?**

a) Yes 60.87%

b) No 39.13%

c) Abstain

**16-revoted) Do you recommend neoadjuvant treatment in patients with solitary kidney and NOT candidate to partial nephrectomy?**

**a) Yes 82%**

b) No 18%

c) Abstain

**17) When you recommend neoadjuvant treatment, do you recommend?**

a) TKI 18.52%

b) ICI + ICI 11.11%

c) ICI + TKI 70.37%

d) Abstain

**17-revoted) When you recommend neoadjuvant treatment, do you recommend?**

a) TKI 3%

b) ICI + ICI 0%

**c) ICI + TKI 97%**

d) Abstain

**18) When you recommend neoadjuvant treatment with TKI, do you recommend?**

a) Sunitinib 34.15%

b) Pazopanib 9.76%

c) Axitinib 24.39%

d) Cabozantinib 31.71%

e) Abstain

**18-revoted) When you recommend neoadjuvant treatment with TKI, do you recommend?**

a) Sunitinib 0%

b) Pazopanib 0%

c) Axitinib 10%

**d) Cabozantinib 48%**

e) No preference 43%

f) Abstain

**19) When you recommend neoadjuvant treatment with ICI + TKI, do you recommend?**

a) Pembrolizumab + Axitinib 28.26%

b) Avelumab + Axitinib 2.17%

c) Nivolumab + Cabozantinib 30.43%

d) No preference 8.70%

e) No preference between combos with overall survival advantage 30.43%

f) Abstain

**19-revoted) When you recommend neoadjuvant treatment with ICI + TKI, do you recommend?**

a) Pembrolizumab + Axitinib 0%

b) Pembrolizumab + Levantinib 32%

c) Avelumab + Axitinib 0%

c) Nivolumab + Cabozantinib 4%

d) No preference 8%

**e) No preference between combos with overall survival advantage 56%**

f) Abstain

**20) Can neoadjuvant therapy be discussed as an exception in initially unresectable cases?**

**a) Yes 94.74%**

b) No 5.26%

c) Abstain

**21) You use indicate neoadjuvant therapy be discussed as an exception in initially unresectable cases, what are you option?**

a) Yes TKIs 16.67%

b) Yes IO 13.33%

c) Yes TKY+IO 56.67%

d) Yes IO+IO 10%

e) No 3.33%

f) Abstain

**21-revoted) You use indicate neoadjuvant therapy be discussed as an exception in initially unresectable cases, what are you option?**

a) Yes TKIs 41%

b) Yes IO 0%

**c) Yes TKY+IO 48%**

d) Yes IO+IO 3%

e) No 7%

f) Abstain

**Advanced renal cell carcinoma management**

**cc Rcc First-line treatment**

**22) Do you consider IMDC risk classification important to define the first-line systemic treatment of metastatic clear cell carcinoma?**

**a) Yes 100%**

b) No 0%

c) Abstain

**23) Do you consider PD-L1 expression important to define the first-line systemic treatment of metastatic clear cell carcinoma?**

a) Yes 0%

**b) No 100%**

c) Abstain

**24) Which treatment do you recommend for first line in patients with metastatic clear cell carcinoma and IMDC favorable risk?**

**a) ICI based 91.18%**

b) High dose IL-2 0%

c) TKI (sunitinib/pazopanib) 8.82%

d) Abstain

**25) When you recommend ICI based treatment for first line in patients with metastatic clear cell carcinoma and IMDC favorable risk, which do you recommend?**

a) ICI + ICI 11.76%

**b) ICI + TKI 82.35%**

c) No preference 5.88%

d) Abstain

**26) Which treatment do you recommend for first line in patients with metastatic clear cell carcinoma and IMDC intermediate risk?**

**a) ICI based 100%**

b) High dose IL-2 0%

c) Abstain

**27) When you recommend ICI based treatment for first line in patients with metastatic clear cell carcinoma and IMDC intermediate risk, which do you recommend?**

a) ICI + ICI 35.29%

b) ICI + TKI 20.59%

c) No preference 44.12%

d) Abstain

**27-revoted) When you recommend ICI based treatment for first line in patients with metastatic clear cell carcinoma and IMDC intermediate risk, which do you recommend?**

a) ICI + ICI 29%

b) ICI + TKI 5%

**c) No preference 67%**

d) Abstain

**28) Which treatment do you recommend for first line in patients with metastatic clear cell carcinoma and IMDC poor risk?**

**a) ICI based 100%**

b) High dose IL-2 0%

c) Abstain

**29) When you recommend ICI based treatment for first line in patients with metastatic clear cell carcinoma and IMDC poor risk, which do you recommend?**

a) ICI + ICI 29.41%

b) ICI + TKI 35.29%

c) No preference 35.29%

d) Abstain

**29-revoted) When you recommend ICI based treatment for first line in patients with metastatic clear cell carcinoma and IMDC poor risk, which do you recommend?**

a) ICI + ICI 18%

b) ICI + TKI 23%

**c) No preference 59%**

d) Abstain

**30) Which treatment do you recommend in patients with metastatic clear cell carcinoma and high burden/symptomatic disease?**

a) ICI + ICI 2.94%

**b) ICI + TKI 97.06%**

c) Abstain

**31) Which treatment do you recommend in patients with metastatic clear cell carcinoma and sarcomatoid/rabdoid features?**

a) ICI + ICI 73.53%

b) ICI + TKI 5.88%

c) No preference between ICI combos (ICI + ICI or ICI + TKI) 20.59%

d) Abstain

**31-revoted) Which treatment do you recommend in patients with metastatic clear cell carcinoma and sarcomatoid/rabdoid features?**

**a) ICI + ICI 77%**

b) ICI + TKI 0%

c) No preference between ICI combos (ICI + ICI or ICI + TKI) 23%

d) TKI (sunitinib/pazopanib/cabozatinib) 0%

e) Temsirolimus 0%

d) Abstain

**32) When you recommend a TKI for first line in patients with IMDC favorable risk metastatic clear cell carcinoma, which do you recommend?**

a) Sunitinib 14.71%

b) Pazopanib 55.88%

c) No preference 29.41%

d) Abstain

**32-revoted) When you recommend a TKI for first line in patients with IMDC favorable risk metastatic clear cell carcinoma, which do you recommend?**

a) Sunitinib 9%

**b) Pazopanib 45%**

**c) No preference 45%**

d) Abstain

**33) When you recommend a TKI for first line in patients with IMDC intermediate/poor-risk metastatic clear cell carcinoma, which do you recommend?**

a) Sunitinib 3.13%

b) Pazopanib 9.38%

**c) Cabozantinib 84.38%**

d) No preference 3.13%

d) Abstain

**34) When you recommend an ICI + TKI combo for first line in patients with metastatic clear cell carcinoma, which do you recommend?**

a) Pembrolizumab + Axitinib 11.76%

b) Pembrolizumab + Levantinib 0%

c) Nivolumab + Cabozantinib 23.53%

d) No preference 5.88%

e) No preference between combos with OS advantage 58.82%

f) Abstain

**34-revoted) When you recommend an ICI + TKI combo for first line in patients with metastatic clear cell carcinoma, which do you recommend?**

a) Pembrolizumab + Axitinib 5%

b) Pembrolizumab + Levantinib 0%

c) Avelumab + Axitinib 0%

d) Nivolumab + Cabozantinib 14%

e) No preference 0%

**f) No preference between combos with OS advantage 81%**

g) Abstain

**Progression after adjuvant treatment**

**35) Which treatment do you recommend for first line in patients with metastatic clear cell carcinoma and IMDC favorable risk, progressing > 12 months after adjuvant pembrolizumab?**

**a) ICI based 94.12%**

b) High dose IL-2 0%

c) TKI 5.88%

d) Abstain

**36) When you recommend ICI based treatment for first line in patients with metastatic clear cell carcinoma and IMDC favorable risk, progressing > 12 months after adjuvant pembrolizumab, which do you recommend?**

a) ICI + ICI 14.71%

**b) ICI + TKI 76.47%**

c) No preference 8.82%

d) Abstain

**37) Which treatment do you recommend for first line in patients with metastatic clear cell carcinoma and IMDC intermediate risk, progressing within 6 months after adjuvant pembrolizumab?**

a) ICI based 32.35%

b) High dose IL2 0%

c) TKI 67.65%

d) Abstain

**37-revoted) Which treatment do you recommend for first line in patients with metastatic clear cell carcinoma and IMDC intermediate risk, progressing within 6 months after adjuvant pembrolizumab?**

a) ICI based 25%

b) High dose IL2 0%

**c) TKI 75%**

d) Abstain

**38) When you recommend ICI based treatment for first line in patients with metastatic clear cell carcinoma and IMDC intermediate risk, progressing within 6 months after adjuvant pembrolizumab, which do you recommend?**

a) ICI + ICI 0%

**b) ICI + TKI 96.55%**

c) No preference 3.45%

d) Abstain

**39) When you recommend a TKI for first line in patients with metastatic clear cell carcinoma and IMDC intermediate risk, progressing within 6 months after adjuvant pembrolizumab, which do you recommend?**

a) Sunitinib 0%

b) Pazopanib 5.88%

c) Cabozantinib 73.53%

d) Lenvatinib + Everolimus 11.76%

e) No preference 8.82%

f) Abstain

**39-revoted) When you recommend a TKI for first line in patients with metastatic clear cell carcinoma and IMDC intermediate risk, progressing within 6 months after adjuvant pembrolizumab, which do you recommend?**

a) Sunitinib 0%

b) Pazopanib 0%

**c) Cabozantinib 95%**

d) Lenvatinib + Everolimus 0%

e) No preference 5%

f) Abstain

**40) Which treatment do you recommend for first line in patients with metastatic clear cell carcinoma and IMDC intermediate risk, progressing > 6 months and < 12 months after adjuvant pembrolizumab?**

a) ICI based 63.64%

b) High dose IL2 0%

c) TKI 36.36%

d) Abstain

**40-revoted) Which treatment do you recommend for first line in patients with metastatic clear cell carcinoma and IMDC intermediate risk, progressing > 6 months and < 12 months after adjuvant pembrolizumab?**

**a) ICI based 68%**

b) High dose IL2 0%

c) TKI 32%

d) Abstain

**41) When you recommend ICI based treatment for first line in patients with metastatic clear cell carcinoma and IMDC intermediate risk, progressing > 6 months and < 12 months after adjuvant pembrolizumab, which do you recommend?**

a) ICI + ICI 3.23%

**b) ICI + TKI 93.55%**

c) No preference 3.23%

d) Abstain

**42) When you recommend a TKI for first line in patients with metastatic clear cell carcinoma and IMDC intermediate risk, progressing > 6 months and < 12 months after adjuvant pembrolizumab, which do you recommend?**

a) Sunitinib 0%

b) Pazopanib 5.88%

**c) Cabozantinib 79.41%**

d) Lenvatinib + Everolimus 5.88%

e) No preference 8.82%

f) Abstain

**43) Which treatment do you recommend for first line in patients with metastatic clear cell carcinoma and IMDC intermediate risk, progressing > 12 months after adjuvant pembrolizumab?**

**a) ICI based 100%**

b) High dose IL-2 0%

c) Abstain

**44) When you recommend ICI based treatment for first line in patients with metastatic clear cell carcinoma and IMDC intermediate risk, progressing > 12 months after adjuvant pembrolizumab, which do you recommend?**

a) ICI + ICI 20.59%

b) ICI + TKI 55.88%

c) No preference 23.53%

d) Abstain

**44-revoted) When you recommend ICI based treatment for first line in patients with metastatic clear cell carcinoma and IMDC intermediate risk, progressing > 12 months after adjuvant pembrolizumab, which do you recommend?**

a) ICI + ICI 14%

b) ICI + TKI 23%

**c) No preference 64%**

d) Abstain

**45) When you recommend a TKI for first line in patients with metastatic clear cell carcinoma and IMDC intermediate risk, progressing > 12 months after adjuvant pembrolizumab, which do you recommend?**

a) Sunitinib 0%

b) Pazopanib 6.06%

**c) Cabozantinib 78.79%**

d) Lenvatinib + Everolimus 9.09%

e) No preference 6.06%

f) Abstain

**46) Which treatment do you recommend for first line in patients with metastatic clear cell carcinoma and IMDC poor-risk, progressing within 6 months after adjuvant pembrolizumab?**

a) ICI based 41.18%

b) High dose IL2 0%

c) TKI 58.82%

d) Abstain

**46-revoted) Which treatment do you recommend for first line in patients with metastatic clear cell carcinoma and IMDC poor risk, progressing within 6 months after adjuvant pembrolizumab?**

a) ICI based 32%

b) High dose IL2 0%

**c) TKI 68%**

d) Abstain

**47) When you recommend ICI based treatment for first line in patients with metastatic clear cell carcinoma and IMDC poor risk, progressing within 6 months after adjuvant pembrolizumab, which do you recommend?**

a) ICI + ICI 3.33%

**b) ICI + TKI 90%**

c) No preference 6.67%

d) Abstain

**48) When you recommend a TKI for first line in patients with metastatic clear cell carcinoma and IMDC poor risk, progressing within 6 months after adjuvant pembrolizumab, which do you recommend?**

a) Sunitinib 0%

b) Pazopanib 2.94%

**c) Cabozantinib 82.35%**

d) Lenvatinib + Everolimus 11.76%

e) No preference 2.94%

f) Abstain

**49) Which treatment do you recommend for first line in patients with metastatic clear cell carcinoma and IMDC poor risk, progressing > 6 months and < 12 months after adjuvant pembrolizumab?**

a) ICI based 61.76%

b) High dose IL-2 0%

c) TKI 38.24%

d) Abstain

**49-revoted) Which treatment do you recommend for first line in patients with metastatic clear cell carcinoma and IMDC poor risk, progressing > 6 months and < 12 months after adjuvant pembrolizumab?**

**a) ICI based 75%**

b) High dose IL-2 0%

c) TKI 25%

d) Abstain

**50) When you recommend ICI based treatment for first line in patients with metastatic clear cell carcinoma and IMDC poor risk, progressing > 6 months and < 12 months after adjuvant pembrolizumab, which do you recommend?**

a) ICI + ICI 0%

**b) ICI + TKI 96.88%**

c) No preference 3.13%

d) Abstain

**51) When you recommend a TKI for first line in patients with metastatic clear cell carcinoma and IMDC poor risk, progressing > 6 months and < 12 months after adjuvant pembrolizumab, which do you recommend?**

a) Sunitinib 0%

b) Pazopanib 2.94%

**c) Cabozantinib 79.41%**

d) Lenvatinib + Everolimus 11.76%

e) No preference 5.88%

f) Abstain

**52) Which treatment do you recommend for first line in patients with metastatic clear cell carcinoma and IMDC poor risk, progressing > 12 months after adjuvant pembrolizumab?**

**a) ICI based 97.06%**

b) High dose IL-2 0%

c) TKI 2.94%

d) Abstain

**53) When you recommend ICI based treatment for first line in patients with metastatic clear cell carcinoma and IMDC poor risk, progressing > 12 months after adjuvant pembrolizumab, which do you recommend?**

a) ICI + ICI 17.65%

b) ICI + TKI 70.59%

c) No preference 11.76%

d) Abstain

**53-revoted) When you recommend ICI based treatment for first line in patients with metastatic clear cell carcinoma and IMDC poor risk, progressing > 12 months after adjuvant pembrolizumab, which do you recommend?**

a) ICI + ICI 14%

**b) ICI + TKI 55%**

c) No preference 32%

d) Abstain

**54) When you recommend a TKI for first line in patients with metastatic clear cell carcinoma and IMDC poor risk, progressing > 12 months after adjuvant pembrolizumab, which do you recommend?**

a) Sunitinib 0%

b) Pazopanib 3.03%

**c) Cabozantinib 81.82%**

d) Lenvatinib + Everolimus 12.12%

e) No preference 3.03%

f) Abstain

**55) Which treatment do you recommend for first line in patients with metastatic clear cell carcinoma and IMDC favorable risk, progressing > 12 months after adjuvant sunitinib?**

**a) ICI based 96.88%**

b) High dose IL-2 0%

c) TKI 3.13%

d) Abstain

**56) When you recommend ICI based treatment for first line in patients with metastatic clear cell carcinoma and IMDC favorable risk, progressing > 12 months after adjuvant sunitinib, which do you recommend?**

a) ICI + ICI 12.50%

b) ICI + TKI 65.63%

c) No preference 21.88%

d) Abstain

**56-revoted) When you recommend ICI based treatment for first line in patients with metastatic clear cell carcinoma and IMDC favorable risk, progressing > 12 months after adjuvant sunitinib, which do you recommend?**

a) ICI + ICI 15%

b) ICI + TKI 30%

**c) No preference 55%**

d) Abstain

**57) Which treatment do you recommend for first line in patients with metastatic clear cell carcinoma and IMDC intermediate risk, progressing within 6 months after adjuvant sunitinib?**

**a) ICI based 96.77%**

b) High dose IL-2 0%

c) TKI 3.23%

d) Abstain

**58) When you recommend ICI based treatment for first line in patients with metastatic clear cell carcinoma and IMDC intermediate risk, progressing within 6 months after adjuvant sunitinib, which do you recommend?**

a) ICI + ICI 67.74%

b) ICI + TKI 16.13%

c) No preference 16.13%

d) Abstain

**58-revoted) When you recommend ICI based treatment for first line in patients with metastatic clear cell carcinoma and IMDC intermediate risk, progressing within 6 months after adjuvant sunitinib, which do you recommend?**

a) ICI + ICI 48%

b) ICI + TKI 0%

**c) No preference 52%**

d) Abstain

**59) When you recommend a TKI for first line in patients with metastatic clear cell carcinoma and IMDC intermediate risk, progressing within 6 months after adjuvant sunitinib, which do you recommend?**

a) Sunitinib 0%

b) Pazopanib 3.33%

**c) Cabozantinib 80%**

d) Lenvatinib + Everolimus 10%

e) No preference 6.67%

f) Abstain

**60) Which treatment do you recommend for first line in patients with metastatic clear cell carcinoma and IMDC intermediate risk, progressing > 6 months and < 12 months after adjuvant sunitinib?**

**a) ICI based 100%**

b) High dose IL-2 0%

c) Abstain

**61) When you recommend ICI based treatment for first line in patients with metastatic clear cell carcinoma and IMDC intermediate risk, progressing > 6 months and < 12 months after adjuvant sunitinib, which do you recommend?**

a) ICI + ICI 58.06%

b) ICI + TKI 25.81%

c) No preference 16.13%

d) Abstain

**61-revoted) When you recommend ICI based treatment for first line in patients with metastatic clear cell carcinoma and IMDC intermediate risk, progressing > 6 months and < 12 months after adjuvant sunitinib, which do you recommend?**

a) ICI + ICI 15%

b) ICI + TKI 5%

**c) No preference 80%**

d) Abstain

**62) When you recommend a TKI for first line in patients with metastatic clear cell carcinoma and IMDC intermediate risk, progressing > 6 months and < 12 months after adjuvant sunitinib, which do you recommend?**

a) Sunitinib 0%

b) Pazopanib 3.33%

**c) Cabozantinib 80%**

d) Lenvatinib + Everolimus 10%

e) No preference 6.67%

f) Abstain

**63) Which treatment do you recommend for first line in patients with metastatic clear cell carcinoma and IMDC intermediate risk, progressing > 12 months after adjuvant sunitinib?**

**a) ICI based 100%**

b) High dose IL-2 0%

c) Abstain

**64) When you recommend a TKI for first line in patients with metastatic clear cell carcinoma and IMDC intermediate risk, progressing > 12 months after adjuvant sunitinib, which do you recommend?**

a) Sunitinib 0%

b) Pazopanib 6.67%

**c) Cabozantinib 80%**

d) Lenvatinib + Everolimus 10%

e) No preference 3.33%

f) Abstain

**65) Which treatment do you recommend for first line in patients with metastatic clear cell carcinoma and IMDC poor risk, progressing within 6 months after adjuvant sunitinib?**

**a) ICI based 100%**

b) High dose IL-2 0%

c) Abstain

**66) When you recommend ICI based treatment for first line in patients with metastatic clear cell carcinoma and IMDC poor risk, progressing within 6 months after adjuvant sunitinib, which do you recommend?**

a) ICI + ICI 61.29%

b) ICI + TKI 19.35%

c) No preference 19.35%

d) Abstain

**66-revoted) When you recommend ICI based treatment for first line in patients with metastatic clear cell carcinoma and IMDC poor risk, progressing within 6 months after adjuvant sunitinib, which do you recommend?**

a) ICI + ICI 45%

b) ICI + TKI 5%

**c) No preference 50%**

d) Abstain

**67) When you recommend a TKI for first line in patients with metastatic clear cell carcinoma and IMDC poor risk, progressing within 6 months after adjuvant sunitinib, which do you recommend?**

a) Sunitinib 0%

b) Pazopanib 3.33%

**c) Cabozantinib 80%**

d) Lenvatinib + Everolimus 10%

e) No preference 6.67%

f) Abstain

**68) Which treatment do you recommend for first line in patients with metastatic clear cell carcinoma and IMDC poor risk, progressing > 6 months and < 12 months after adjuvant sunitinib?**

**a) ICI based 100%**

b) High dose IL-2 0%

c) TKI 0%

d) Abstain

**69) When you recommend ICI based treatment for first line in patients with metastatic clear cell carcinoma and IMDC poor risk, progressing > 6 months and < 12 months after adjuvant sunitinib, which do you recommend?**

a) ICI + ICI 63.33%

b) ICI + TKI 23.33%

c) No preference 13.33%

d) Abstain

**69-revoted) When you recommend ICI based treatment for first line in patients with metastatic clear cell carcinoma and IMDC poor risk, progressing > 6 months and < 12 months after adjuvant sunitinib, which do you recommend?**

a) ICI + ICI 14%

b) ICI + TKI 10%

**c) No preference 76%**

d) Abstain

**70) When you recommend a TKI for first line in patients with metastatic clear cell carcinoma and IMDC poor risk, progressing > 6 months and < 12 months after adjuvant sunitinib, which do you recommend?**

a) Sunitinib 0%

b) Pazopanib 3.33%

**c) Cabozantinib 80%**

d) Lenvatinib + Everolimus 10%

e) No preference 6.67%

f) Abstain

**71) Which treatment do you recommend for first line in patients with metastatic clear cell carcinoma and IMDC poor risk, progressing > 12 months after adjuvant sunitinib?**

**a) ICI based 100%**

b) High dose IL-2 0%

c) TKI 0%

d) Abstain

**72) When you recommend ICI based treatment for first line in patients with metastatic clear cell carcinoma and IMDC poor risk, progressing > 12 months after adjuvant sunitinib, which do you recommend?**

a) ICI + ICI 41.94%

b) ICI + TKI 32.26%

c) No preference 25.81%

d) Abstain

**72-revoted) When you recommend ICI based treatment for first line in patients with metastatic clear cell carcinoma and IMDC poor risk, progressing > 12 months after adjuvant sunitinib, which do you recommend?**

a) ICI + ICI 9%

b) ICI + TKI 9%

**c) No preference 82%**

d) Abstain

**73) When you recommend a TKI for first line in patients with metastatic clear cell carcinoma and IMDC poor risk, progressing > 12 months after adjuvant sunitinib, which do you recommend?**

a) Sunitinib 0%

b) Pazopanib 3.33%

**c) Cabozantinib 86.67%**

d) Lenvatinib + Everolimus 6.67%

e) No preference 3.33%

f) Abstain

**ccRCC Second line therapy**

**74) With the current data of combinations of antiangiogenic and immunotherapy (ICI), or ICI-ICI therapy, I believe that high-dose IL2 therapy:**

**a) No longer has a role in treatment 91.18%**

b) Has some role in selected cases 8.82%

c) Abstain

**75) For patients progressing after immunotherapy combo (Nivo + Ipi) in first line, which treatment do you recommend?**

**a) TKI 94.12%**

b) Everolimus 0%

c) Lenvatinib + Everolimus 5.88%

d) Abstain

**76) When you recommend a TKI for patients progressing after immunotherapy combo (Nivo + Ipi) in 1st line, which treatment do you recommend?**

a) Sunitinib 0%

b) Pazopanib 0%

c) Axitinib 2.94%

**d) Cabozantinib 88.24%**

e) Sorafenib 0%

f) No preference 8.82%

g) Abstain

**77) For patients progressing after ICI + TKI in first line, which treatment do you recommend?**

**a) TKI not used previously 76.47%**

b) Everolimus 0%

c) Lenvatinib + Everolimus 23.53%

d) Abstain

**78) For patients progressing after TKI in first line, which treatment do you recommend?**

a) Axitinib 0%

b) Cabozantinib 23.53%

c) Nivolumab 61.76%

d) Everolimus 0%

e) Lenvatinib + Everolimus 5.88%

f) Sorafenib 0%

g) No preference 8.82%

h) Abstain

**78-revoted) For patients progressing after TKI in first line, which treatment do you recommend?**

a) Axitinib 0%

b) Cabozantinib 11%

**c) Nivolumab 74%**

d) Everolimus 0%

e) Lenvatinib + Everolimus 0%

f) Sorafenib 0%

g) No preference 16%

h) Abstain

**79) For patients progressing after TKI in first line, do you prefer Nivolumab?**

**a) In a majority of cases 91.18%**

b) In a minority of cases 8.82%

c) Abstain

**80) For patients progressing after TKI (sunitinib/pazopanib) in first line, do you prefer Cabozantinib?**

a) In a majority of cases 35.29%

b) In a minority of cases 64.71%

c) Abstain

**80-revoted) For patients progressing after TKI (sunitinib/pazopanib) in first line, do you prefer Cabozantinib?**

a) In a majority of cases 15%

**b) In a minority of cases 85%**

c) Abstain

**ccRCC Third-line therapy**

**81) For patients who progressed after combo immunotherapy treatment (ICI + ICI or ICI + TKI) and a TKI in 2nd line, which treatment do you recommend?**

a) TKI not previously used 23.53%

b) Everolimus 0%

**c) Lenvatinib + Everolimus 76.47%**

d) Abstain

**82) For patients who progressed after treatment with cabozantinib (1st line) and immunotherapy (Nivo in 2nd line), which treatment do you recommend?**

a) TKI not previously used 11.76%

b) Everolimus 5.88%

**c) Lenvatinib + Everolimus 82.35%**

d) Abstain

**83) For patients who progressed after treatment with VEGF inhibitors and everolimus, which treatment do you recommend?**

a) TKI not previously used 20.59%

**b) Nivolumab 79.41%**

c) Abstain

**First-line treatment for metastatic disease (non-cc histology)**

**84) Which treatment do you recommend for the majority of papillary tumors?**

a) Sunitinib 5.88%

b) Cabozantinib 64.71%

c) Everolimus 0%

d) Bevacizumab + Erlotinib 2.94%

e) ICI + TKI 26.47%

f) Abstain

**84-revoted) Which treatment do you recommend for the majority of papillary tumors?**

a) Sunitinib 0%

**b) Cabozantinib 60%**

c) Everolimus 0%

d) Bevacizumab + Erlotinib 5%

e) ICI + TKI 35%

f) Abstain

**85) Which treatment do you recommend for the majority of fumarate hydratase mutant tumors?**

a) Sunitinib 6.25%

b) Cabozantinib 21.88%

c) Everolimus 0%

d) Bevacizumab + Erlotinib 62.50%

e) ICI + TKI 9.38%

f) Abstain

**85-revoted) Which treatment do you recommend for the majority of fumarate hydratase mutant tumors?**

a) Sunitinib 0%

b) Cabozantinib 0%

c) Everolimus 0%

**d) Bevacizumab + Erlotinib 100%**

e) ICI + TKI 0 %

f) Abstain

**86) Which treatment do you recommend for the majority of unclassified tumors?**

a) Sunitinib 9.09%

b) Cabozantinib 15.15%

c) Everolimus 0%

d) ICI + ICI 18.18%

e) ICI + TKI 57.58%

f) Abstain

**86-revoted) Which treatment do you recommend for the majority of unclassified tumors?**

a) Sunitinib 10%

b) Cabozantinib 5%

c) Everolimus 0%

d) ICI + ICI 0%

**e) ICI + TKI 86%**

f) Abstain

**87) Which treatment do you recommend for the majority of chromophobe tumors?**

a) Sunitinib 15.15%

b) Cabozantinib 24.24%

c) Everolimus 9.09%

d) Lenvatinib + Everolimus 27.27%

e) ICI + TKI 24.24%

f) Abstain

**87-revoted) Which treatment do you recommend for the majority of chromophobe tumors?**

a) Sunitinib 5%

b) Cabozantinib 0%

c) Everolimus 10%

**d) Lenvatinib + Everolimus 86%**

e) ICI + TKI 0%

f) Abstain

**88) Which treatment do you recommend for the majority of MiT family translocations tumors?**

a) Sunitinib 18.18%

b) Cabozantinib 24.24%

c) Everolimus 0%

d) ICI + ICI 9.09%

e) ICI + TKI 48.48%

f) Abstain

**88-revoted) Which treatment do you recommend for the majority of MiT family translocations tumors?**

a) Sunitinib 5%

b) Cabozantinib 5%

c) Everolimus 0%

d) ICI + ICI 0%

**e) ICI + TKI 90%**

f) Abstain

**89) Which treatment do you recommend for the majority of collecting ducts tumors?**

a) TKI 0%

b) ICI + ICI 0%

c) ICI + TKI 0%

**d) Platinum-based chemotherapy 100%**

f) Abstain

**90) Which treatment do you recommend for the majority of medullary tumors?**

a) TKI 8.82%

b) ICI + ICI 0%

c) ICI + TKI 2.94%

**d) Platinum-based chemotherapy 88.24%**

f) Abstain

**91) Do you change the treatment if the percentage of sarcomatoid pattern is > 20%?**

a) Yes 32.26%

b) No 67.74%

c) Abstain

**91-revoted) Do you change the treatment if the percentage of sarcomatoid pattern is > 20%?**

a) Yes 26%

**b) No 74%**

c) Abstain

**92) Which treatment do you recommend for tumors with sarcomatoid pattern?**

a) TKI 0%

b) Sunitinib + gemcitabine 0%

c) Gemcitabine + doxorubicin 0%

**d) ICI + ICI 81.25%**

e) ICI + TKI 18.75%

f) Abstain

**Second-line treatment for metastatic disease (non-cc histology)**

**93) Which treatment do you recommend after TKI for the majority of papillary tumors?**

a) Sunitinib or pazopanib or cabozantinib (not previously used) 29.41%

b) Everolimus 8.82%

c) Bevacizumab + Erlotinib 8.82%

d) Nivolumab 52.94%

e) Abstain

**93-revoted) Which treatment do you recommend after TKI for the majority of papillary tumors?**

a) Sunitinib or pazopanib or cabozantinib (not previously used) 19%

b) Everolimus 0%

**c) Bevacizumab + Erlotinib 69%**

d) Nivolumab 13%

e) Abstain

**94) Which treatment do you recommend after mTOR inhibitors for the majority of papillary tumors?**

a) Sunitinib or pazopanib or cabozantinib (not previously used) 66.67%

b) Bevacizumab + Erlotinib 6.06%

c) Nivolumab 27.27%

d) Abstain

**94-revoted) Which treatment do you recommend after mTOR inhibitors for the majority of papillary tumors?**

**a) Sunitinib or pazopanib or cabozantinib (not previously used) 100%**

b) Bevacizumab + Erlotinib 0%

c) Nivolumab 0%

d) Abstain

**Active surveillance**

**95) Do you recommend active surveillance as part of the strategy of treatment in patients with metastatic clear cell carcinoma and IMDC favorable risk?**

a) Yes, in a majority of patients 17.65%

**b) Yes, in a minority of patients 82.35%**

c) No 0%

d) Abstain

**96) Do you recommend active surveillance as part of the strategy of treatment in patients with metastatic clear cell carcinoma and IMDC intermediate risk?**

a) Yes, in a majority of patients 0%

b) Yes, in a minority of patients 20.59%

**c) No 79.41%**

d) Abstain

**97) Do you recommend active surveillance as part of the strategy of treatment in patients with metastatic clear cell carcinoma and IMDC poor risk?**

a) Yes, in a majority of patients 0%

b) Yes, in a minority of patients 0%

**c) No 100%**

d) Abstain

**98) Do you recommend active surveillance in patients with bone metastasis?**

a) Yes, in a majority of patients 0%

b) Yes, in a minority of patients 38.24%

c) No 61.76%

d) Abstain

**98-revoted) Do you recommend active surveillance in patients with bone metastasis?**

a) Yes, in a majority of patients 0%

b) Yes, in a minority of patients 47%

**c) No 53%**

d) Abstain

**99) Do you recommend active surveillance in patients with lung metastasis?**

a) Yes, in a majority of patients 8.82%

**b) Yes, in a minority of patients 85.29%**

c) No 5.88%

d) Abstain

**100) Do you recommend active surveillance in patients with liver metastasis?**

a) Yes, in a majority of patients 0%

b) Yes, in a minority of patients 5.88%

**c) No 94.12%**

d) Abstain

**101) Do you recommend active surveillance in patients with lymph-node metastasis?**

a) Yes, in a majority of patients 11.76%

**b) Yes, in a minority of patients 82.35%**

c) No 5.88%

d) Abstain

**102) Do you recommend active surveillance in patients with brain metastasis?**

a) Yes, in a majority of patients 0%

b) Yes, in a minority of patients 11.76%

**c) No 88.24%**

d) Abstain

**103) Do you recommend active surveillance in patients with one metastatic site?**

a) Yes, in a majority of patients 23.53%

b) Yes, in a minority of patients 67.65%

c) No 8.82%

d) Abstain

**103-revoted) Do you recommend active surveillance in patients with one metastatic site?**

a) Yes, in a majority of patients 32%

**b) Yes, in a minority of patients 63%**

c) No 5%

d) Abstain

**104) Do you recommend active surveillance in patients with ≥ 2 metastatic sites?**

a) Yes, in a majority of patients 0%

b) Yes, in a minority of patients 58.82%

c) No 41.18%

d) Abstain

**104-revoted) Do you recommend active surveillance in patients with ≥ 2 metastatic sites?**

a) Yes, in a majority of patients 0%

b) Yes, in a minority of patients 21%

**c) No 79%**

d) Abstain

**105) Do you recommend active surveillance in patients with symptoms from the disease?**

a) Yes, in a majority of patients 0%

b) Yes, in a minority of patients 5.88%

**c) No 94.12%**

d) Abstain

**Osteoclast inhibitors**

**106) Which treatment do you recommend for metastatic bone disease?**

a) Zoledronic acid 2.94%

b) Denosumab 61.76%

c) If there is no contraindication to any agent, I have no preference 35.29%

d) Abstain

**106-revoted) Which treatment do you recommend for metastatic bone disease?**

a) Zoledronic acid 0%

b) Denosumab 28%

**c) If there is no contraindication to any agent, I have no preference 72%**

d) Abstain

**107) When you use denosumab 120 mg, which frequency do you recommend?**

**a) Monthly 82.35%**

b) Every 12 weeks 17.65%

c) Abstain

**108) When you use zoledronic acid 4 mg, which frequency do you recommend?**

a) Monthly 32.35%

b) Every 12 weeks 67.65%

c) Abstain

**108-revoted) When you use zoledronic acid 4 mg, which frequency do you recommend?**

a) Monthly 19%

**b) Every 12 weeks 81%**

c) Abstain

**Brain metastasis**

**109) In patients with metastatic clear cell RCC and limited brain metastasis (1-3 lesions), which local therapy do you recommend?**

a) Surgery in the majority of patients 17.65%

**b) SBRT in the majority of patients 82.35%**

c) Abstain

**110) In patients with metastatic clear cell RCC and limited brain metastasis (1-3 lesions), with no/low dose steroids required, which systemic therapy do you recommend?**

a) ICI + ICI 41.18%

b) ICI + TKI 58.82%

c) High dose IL-2 0%

d) TKI (sunitinib/pazopanib) 0%

e) TKI (cabozantinib) 0%

f) Abstain

**110-revoted) In patients with metastatic clear cell RCC and limited brain metastasis (1-3 lesions), with no/low dose steroids required, which systemic therapy do you recommend?**

**a) ICI + ICI 69%**

b) ICI + TKI 31%

c) High dose IL-2 0%

d) TKI (sunitinib/pazopanib) 0%

e) TKI (cabozantinib) 0%

f) Abstain

**111) In patients with metastatic clear cell RCC and several brain metastases, which local therapy do you recommend?**

**a) Whole brain radiotherapy in the majority of patients 94.12%**

b) I do not recommend local therapy in the majority of patients 5.88%

c) Abstain

**112) In patients with metastatic clear cell RCC and several brain metastases, with no/low dose steroids required, which systemic therapy do you recommend?**

a) ICI + ICI 29.41%

b) ICI + TKI 70.59%

c) High dose IL-2 0%

d) TKI (sunitinib/pazopanib) 0%

e) TKI (cabozantinib) 0%

f) Abstain

**112-revoted) In patients with metastatic clear cell RCC and several brain metastases, with no/low dose steroids required, which systemic therapy do you recommend?**

**a) ICI + ICI 63%%**

b) ICI + TKI 38%

c) High dose IL-2 0%

d) TKI (sunitinib/pazopanib) 0%

e) TKI (cabozantinib) 0%

f) Abstain
